# Mutations in the Key Autophagy Tethering Factor EPG5 Link Neurodevelopmental and Neurodegenerative Disorders Including Early‐Onset Parkinsonism

**DOI:** 10.1002/ana.78013

**Published:** 2025-10-06

**Authors:** Hormos Salimi Dafsari, Celine Deneubourg, Kritarth Singh, Reza Maroofian, Zita Suprenant, Ay Lin Kho, Neil J Ingham, Karen P Steel, Preethi Sheshadri, Franciska Baur, Lea Hentrich, Birgit Gerisch, Mina Zamani, Cesar Alves, Ata Siddiqui, Haidar S Dafsari, Mehri Salari, Anthony E. Lang, Michael Harris, Alice Abdelaleem, Saeid Sadeghian, Reza Azizimalamiri, Hamid Galehdari, Gholamreza Shariati, Alireza Sedaghat, Jawaher Zeighami, Daniel Calame, Dana Marafi, Ruizhi Duan, Adrian Boehnke, Gary D. Clark, Jill A. Rosenfeld, Carrie A. Mohila, Dora Steel, Saurabh Chopra, Suvasini Sharma, Nicolai Kohlschmidt, Steffi Patzer, Afshin Saffari, Darius Ebrahimi‐Fakhari, Büşra Eser Çavdartepe, Irene J Chang, Erika Beckman, Renate Peters, Andrew Paul Fennell, Bernice Lo, Luisa Averdunk, Felix Distelmaier, Martina Baethmann, Frances Elmslie, Kairit Joost, Sheela Nampoothiri, Dhanya Yesodharan, Hanna Mandel, Amy Kimball, Antonie D. Kline, Cyril Mignot, Boris Keren, Vincent Laugel, Katrin Õunap, Kalpana Devadathan, Frederique M.C. van Berkestijn, Arpana Silwal, Saskia Koene, Sumit Verma, Mohammed Yousuf Karim, Chahynez Boubidi, Majid Aziz, Gehad ElGhazali, Lauren Mattas, Mohammad Miryounesi, Farzad Hashemi‐Gorji, Shahryar Alavi, Nayereh Nouri, Mehrdad Noruzinia, Saeideh Kavousi, Arveen Kamath, Sandeep Jayawant, Russell Saneto, Nourelhoda A. Haridy, Pinar Ozkan Kart, Ali Cansu, Madeleine Joubert, Claire Beneteau, Kyra E. Stuurman, Martina Wilke, Tahsin Stefan Barakat, Homa Tajsharghi, Annarita Scardamaglia, Sadeq Vallian, Semra Hız, Ali Shoeibi, Reza Boostani, Narges Hashemi, Meisam Babaei, Norah Saleh Alsaleh, Julie Porter, Tania Attié‐Bitach, Pauline Marzin, Dorota Wicher, Jessica I. Gold, Elisabeth Schuler, Amna Kashgari, Rakan F. Alanazi, Wafaa Eyaid, Marc Engelen, Mirjam Langeveld, Burkhard Stüve, Yun Li, Gökhan Yigit, Bernd Wollnik, Mariana H.G Monje, Dimitri Krainc, Niccolò E. Mencacci, Somayeh Bakhtiari, Michael Kruer, Emanuela Argilli, Elliott Sherr, Yalda Jamshidi, Ehsan Ghayoor Karimiani, Yiu Wing Sunny Cheung, Ivan Karin, Giovanni Zifarelli, Peter Bauer, Wendy K Chung, James R. Lupski, Manju A. Kurian, Jörg Dötsch, Jürgen‐Christoph von Kleist‐Retzow, Thomas Klopstock, Matias Wagner, Calvin Yip, Andreas Roos, Rita Carsetti, Carlo Dionisi‐Vici, Mathias Gautel, Michael R Duchen, Adam Antebi, Henry Houlden, Manolis Fanto, Heinz Jungbluth

**Affiliations:** ^1^ Department of Pediatrics and Center for Rare Diseases, Faculty of Medicine and University Hospital Cologne University of Cologne Cologne Germany; ^2^ Max Planck Institute for Biology of Aging and Cologne Excellence Cluster for Aging‐associated Diseases Cologne Germany; ^3^ Department of Pediatric Neurology Evelina London Children's Hospital, Guy's & St Thomas NHS Foundation Trust London UK; ^4^ Randall Center for Cell and Molecular Biophysics, Muscle Signaling Section, Faculty of Life Sciences and Medicine (FoLSM) King's College London London UK; ^5^ Department of Basic and Clinical Neuroscience, Institute of Psychiatry, Psychology & Neuroscience King's College London London UK; ^6^ UCL Consortium for Mitochondrial Research and Department of Cell and Developmental Biology University College London London UK; ^7^ Department of Neuromuscular Diseases UCL Queen Square Institute of Neurology London UK; ^8^ Vici Syndrome Foundation, Inc Silver Spring Maryland USA; ^9^ Wolfson Sensory Pain and Regeneration Centre, Institute of Psychiatry, Psychology & Neuroscience King's College London London UK; ^10^ Narges Medical Genetics and Prenatal Diagnosis Laboratory Ahvaz Iran; ^11^ Department of Biology, Faculty of Science Shahid Chamran University of Ahvaz Ahvaz Iran; ^12^ Department of Radiology Boston Children's Hospital Boston MA USA; ^13^ Department of Radiology Guy's and Saint Thomas' Hospitals NHS Trust London UK; ^14^ Department of Neurology, Faculty of Medicine and University Hospital Cologne University of Cologne Cologne Germany; ^15^ Faculty of Medicine Shahid Beheshti University of Medical Sciences Tehran Iran; ^16^ Edmond J Safra Program in Parkinson's Disease, Krembil Brain Institute, University Health Network and the Department of Medicine University of Toronto Toronto ON Canada; ^17^ Department of Neurology Weill Cornell Medicine Qatar, Education City Doha Qatar; ^18^ Medical Molecular Genetics Institute Human Genetics and Genome Research, National Research Centre Dokki Egypt; ^19^ Department of Neurology University of Minnesota Minneapolis Minnesota USA; ^20^ Department of Medical Genetics, Faculty of Medicine Ahvaz Jundishapur University of Medical Sciences Ahvaz Iran; ^21^ Diabetes Research Center, Health Research Institute Ahvaz Jundishapur University of Medical Sciences Ahvaz Iran; ^22^ Department of Pediatrics Baylor College of Medicine Houston TX USA; ^23^ Department of Molecular and Human Genetics Baylor College of Medicine Houston TX USA; ^24^ Baylor Genetics Laboratories Houston TX USA; ^25^ Department of Pathology Department of Pathology and Immunology Texas Children's Hospital Baylor College of Medicine Houston TX USA; ^26^ Developmental Neurosciences, Zayed Centre for Research into Rare Disease in Children UCL GOS‐Institute of Child Health London UK; ^27^ Indraprastha Apollo Hospital New Delhi India; ^28^ Department of Pediatrics Lady Hardinge Medical College and Associated Kalawati Saran Children's Hospital New Delhi India; ^29^ Institute for Clinical Genetics and Tumour Genetics Bonn Germany; ^30^ Laboratoire national de santé, National Center of Genetics, Dudelange, Luxembourg; ^31^ Department of Pediatrics Krankenhaus St. Elisabeth und St. Barbara Halle (Saale) Germany; ^32^ Division of Child Neurology and Metabolic Medicine, Department of Pediatrics I, Center for Pediatrics and Adolescent Medicine, Medical Faculty Heidelberg, University Hospital Heidelberg Heidelberg University Heidelberg Germany; ^33^ Movement Disorders Program, Department of Neurology, Boston Children's Hospital Harvard Medical School Boston MA USA; ^34^ Department of Medical Genetics Konya City Hospital Konya Turkey; ^35^ Department of Pediatrics, Division of Medical Genetics University of California at San Francisco San Francisco CA USA; ^36^ Division of Genetic Medicine, Department of Pediatrics University of Washington Seattle WA USA; ^37^ Christliches Kinderhospital Osnabrück Osnabrück Germany; ^38^ Monash Genetics Monash Health Melbourne Vic Australia; ^39^ Department of Paediatrics Monash University Melbourne Vic Australia; ^40^ Research Branch, Sidra Medicine, Doha; College of Health and Life Sciences Hamad Bin Khalifa University Doha Qatar; ^41^ Department of General Pediatrics, Neonatology and Pediatric Cardiology, Medical Faculty Heinrich‐Heine‐University Düsseldorf Germany; ^42^ Department of Pediatrics Hospital Dritter Orden Munich Germany; ^43^ St George's University Hospitals NHS Foundation Trust London UK; ^44^ Faculty of Medicine University of Tartu Tartu Estonia; ^45^ Department of Pediatric Genetics Amrita Institute of Medical Sciences & Research Center Cochin India; ^46^ Department of Metabolic and Genetic Disorders Ziv, Medical Center Safed Israel; ^47^ Harvey Institute for Human Genetics, Greater Baltimore Medical Center Baltimore MD USA; ^48^ APHP, Hôpital Pitié‐Salpêtrière, Département de Génétique, Centre de Reference Déficience Intellectuelle de Causes Rares GRC UPMC Déficience Intellectuelle et Autisme Paris France; ^49^ Service de Pédiatrie Centre Hospitalier Universitaire (CHU) de Strasbourg‐Hautepierre Strasbourg France; ^50^ Department of Clinical Genetics, Genetics and Personalized Medicine Clinic, Tartu University Hospital and Institute of Clinical Medicine University of Tartu Tartu Estonia; ^51^ Department of Pediatric Neurology Government Medical College Thiruvananthapuram India; ^52^ Department of Pediatric Neurology University Medical Center Utrecht Utrecht the Netherlands; ^53^ St Bart's Health NHS Trust London UK; ^54^ Department of Clinical Genetics Leiden University Medical Center Leiden the Netherlands; ^55^ Children's Healthcare of Atlanta Emory University Atlanta GA USA; ^56^ Department of Pathology, Sidra Medicine College of Medicine, Qatar University Doha Qatar; ^57^ Department of Pediatrics A, Hussein Dey University Hospital Center University of Algiers 1 Algiers Algeria; ^58^ Department of Pediatric Neurology Sheikh Khalifa Medical City Abu Dhabi United Arab Emirates; ^59^ Department of Medical Microbiology and Immunology, College of Medicine and Health Sciences United Arab Emirates University Al Ain United Arab Emirates; ^60^ Stanford Children's Hospital Palo Alto CA USA; ^61^ Department of Medical Genetics, Faculty of Medicine Shahid Beheshti University of Medical Sciences Tehran Iran; ^62^ Genomic Research Center Shahid Beheshti University of Medical Sciences Tehran Iran; ^63^ Palindrome Isfahan Iran; ^64^ Karyogen Lab Isfahan Iran; ^65^ Department of Laboratory Medicine and Pathology University of Washington Seattle WA USA; ^66^ Department of Medical Genetics, Faculty of Medical Sciences Tarbiat Modares University Tehran Iran; ^67^ Cardiff and Vale UHB–AWMGS Cardiff UK; ^68^ Oxford University Hospitals NHS Foundation Trust Oxford UK; ^69^ Neuroscience Institute, Center for Integrated Brain Research, Department of Neurology/Division of Pediatric Neurology, Seattle Children's Hospital Seattle WA USA; ^70^ Department of Neurology, Faculty of Medicine Assiut University Assiut Egypt; ^71^ Department of Pediatrics Neurology Karadeniz Technical University Trabzon Turkey; ^72^ Genetics Department Nantes University Hospital Nantes France; ^73^ Department of Clinical Genetics Erasmus MC University Medical Center Rotterdam the Netherlands; ^74^ School of Health Science, Division Biomedicine and Translational Medicine University of Skovde Skovde Sweden; ^75^ Department of Cell and Molecular Biology & Microbiology, Faculty of Science and Technology University of Isfahan Isfahan Iran; ^76^ Faculty of Medicine, Pediatric Neurology Department Dokuz Eylül University Izmir Turkey; ^77^ Department of Neurology Mashhad University of Medical Sciences Mashhad Iran; ^78^ Department of Pediatrics, School of Medicine Mashhad University of Medical Sciences Mashhad Iran; ^79^ Rare Pediatric Neurological Diseases Research Center Mashhad University of Medical Sciences Mashhad Iran; ^80^ Department of Pediatrics North Khorasan University of Medical Sciences Bojnurd Iran; ^81^ Department of Genetics and Precision Medicine King Abdullah Specialized Children's Hospital, King Abdullah International Medical Research Center, Ministry of National Guard Health Affairs Riyadh Saudi Arabia; ^82^ Division of Medical Genetics, Department of Pediatrics University of Utah Salt Lake City UT USA; ^83^ Service de Médecine Génomique des Maladies Rares Hôpital Necker‐Enfants Malades Paris France; ^84^ Department of Medical Genetics Children's Memorial Health Institute Warsaw Poland; ^85^ Division of Medical Genetics, Department of Pediatrics Cohen Children's Medical Center New Hyde Park NY USA; ^86^ Department of Clinical Genetics and Precision Medicine King Abdulaziz Medical City Riyadh Saudi Arabia; ^87^ Amsterdam Leukodystrophy Center, Department of Pediatric Neurology, Emma Children's Hospital, and Amsterdam Neuroscience, Cellular & Molecular Mechanisms, Amsterdam University Medical Center University of Amsterdam Amsterdam the Netherlands; ^88^ Department of Endocrinology and Metabolism, Amsterdam UMC, Research Institute Gastroenterology, Endocrinology & Metabolism (AGEM) University of Amsterdam Amsterdam the Netherlands; ^89^ Department for Neuropediatrics DRK Children's Hospital Siegen Siegen Germany; ^90^ Institute of Human Genetics University Medical Center Göttingen Göttingen Germany; ^91^ DZHK (German Center for Cardiovascular Research) Partner Site Lower Saxony Göttingen Germany; ^92^ Cluster of Excellence “Multiscale Bioimaging: From Molecular Machines To Networks of Excitable Cells” (MBExC) University of Göttingen; German Center for Child and Adolescent Health (DZKJ), Partner Site Göttingen Göttingen Germany; ^93^ Department of Neurology Northwestern University Feinberg School of Medicine Chicago IL USA; ^94^ Pediatric Movement Disorders Program, Division of Pediatric Neurology Barrow Neurological Institute, Phoenix Children's Hospital Phoenix AZ USA; ^95^ Department of Neurology University of California San Francisco Division of Hospital Medicine San Francisco CA USA; ^96^ Molecular and Clinical Sciences Institute St. George's University of London London UK; ^97^ Life Sciences Institute, Department of Biochemistry and Molecular Biology The University of British Columbia Vancouver BC Canada; ^98^ Friedrich‐Baur‐Institute, Department of Neurology, LMU University Hospital Ludwig‐Maximilians‐Universität München Munich Germany; ^99^ CENTOGENE GmbH Rostock Germany; ^100^ Department of Pediatrics Boston Children's Hospital and Harvard Medical School Boston MA USA; ^101^ German Center for Neurodegenerative Diseases (DZNE) Munich Germany; ^102^ Munich Cluster for Systems Neurology (SyNergy) Munich Germany; ^103^ Institute of Human Genetics, School of Medicine and Health Technische Universität München Munich Germany; ^104^ Department of Pediatric Neurology, Centre for Neuromuscular Disorders, Centre for Translational Neuro‐ and Behavioral Sciences University Duisburg‐Essen Essen Germany; ^105^ Department of Neurology, Medical Faculty and University Hospital Düsseldorf Heinrich Heine University Düsseldorf Germany; ^106^ Brain and Mind Research Institute, Children's Hospital of Eastern Ontario Research Institute Ottawa ON Canada; ^107^ Immunology Research Area, B Cell Unit Ospedale Pediatrico Bambino Gesù IRCCS Rome Italy; ^108^ Division of Metabolic Diseases and Hepatology Bambino Gesù Children's Hospital, IRCCS Rome Italy

## Abstract

**Objective:**

Autophagy is a fundamental biological pathway with vital roles in intracellular homeostasis. During autophagy, defective cargoes including mitochondria are targeted to lysosomes for clearance and recycling. Recessive truncating variants in the autophagy gene *EPG5* have been associated with Vici syndrome, a severe early‐onset neurodevelopmental disorder with extensive multisystem involvement. Here, we aimed to delineate the extended, age‐dependent *EPG5*‐related disease spectrum.

**Methods:**

We investigated clinical, radiological, and molecular features from the largest cohort of *EPG5*‐related patients identified to date, complemented by experimental investigation of cellular and animal models of EPG5 defects.

**Results:**

Through worldwide collaboration, we identified 211 patients, 97 of them previously unpublished, with recessive *EPG5* variants. The phenotypic spectrum ranged from antenatally lethal presentations to milder isolated neurodevelopmental disorders. A novel Epg5 knock‐in mouse model of a recurrent *EPG5* missense variant featured motor impairments and defective autophagy in brain areas particularly relevant for the neurological disorders in milder presentations. Novel age‐dependent neurodegenerative manifestations in our cohort included adolescent‐onset parkinsonism and dystonia with cognitive decline, and myoclonus. Radiological features suggested an emerging continuum with brain iron accumulation disorders. Patient fibroblasts showed defects in PINK1‐Parkin‐dependent mitophagic clearance and α‐synuclein overexpression, indicating a cellular basis for the observed neurodegenerative phenotypes. In *Caenorhabditis elegans*, *EPG5* knockdown caused motor impairments, defective mitophagic clearance, and changes in mitochondrial respiration comparable to observations in *C. elegans* knockdown of parkinsonism‐related genes.

**Interpretation:**

Our findings illustrate a lifetime neurological disease continuum associated with pathogenic *EPG5* variants, linking neurodevelopmental and neurodegenerative disorders through the common denominator of defective autophagy. ANN NEUROL 2025;98:932–950

Autophagy is a fundamental biological pathway conserved throughout evolution with important roles in intracellular quality control and homeostasis.^1^ Its most common form, macroautophagy, is characterized by the engulfment of intracellular targets by double‐membraned structures, autophagosomes, and their delivery to the lysosome for digestion and recycling. Autophagy is specifically capable of processing larger protein complexes and intracellular organelles, such as mitochondria (a process termed “mitophagy”).^1^

Autophagy has been implicated non‐specifically in a wide range of human disease, but monogenic primary autophagy defects in humans have only been recognized relatively recently.^2^, ^3^ Vici syndrome (VS), the paradigmatic disorder of defective autophagy characterized by both neurodevelopmental and multisystem features, is caused by recessive variants in *EPG5*, encoding the ectopic P‐granules 5 autophagy protein with a key role in autophagosome–lysosome fusion.^4^, ^5^, ^6^ In one of our previous studies, we observed that heterozygous *EPG5* pathogenic variant carriers may have an increased risk of movement disorders,^7^ indicating a gene dosage effect on EPG5 function. This observation prompted the hypothesis that relatively milder phenotypes, in particular associated with recessive *EPG5* missense variants, may show age‐dependent progression with premature neurodegenerative events.

In this study, we report an expanded age‐dependent phenotypic spectrum of *EPG5*‐related disorders (*EPG5*‐RD), including rapidly progressive adolescent‐onset movement disorders, such as parkinsonism with dystonia and subsequent cognitive decline. These novel age‐related neurological presentations are also supported by complementary experimental findings in mouse, worm, and human cellular models of defective EPG5 function, which, taken together, also suggest more specific pathogenic mechanisms underlying the expanded spectrum of *EPG5*‐related disorders linked to abnormal autophagy.

## Methods

Key methods for the different parts of our study are outlined here, and additional information is detailed in Supplementary File 1.

### Study Approval

The study was approved by regional institutional review boards.^8^, ^9^ All patients and/or their legal guardians gave informed consent to anonymized publication and use of recognizable photographs/videos where applicable.

### Clinical Investigations

Patients with biallelic *EPG5* variants were recruited via established collaborations, web platforms (GeneMatcher, Varsome), and national studies focusing on rare pediatric neurological diseases in the European Union (ERN ITHACA), Germany (ESNEK), and the UK (100K Genomes Project, DDD study).^10^, ^11^, ^12^ The diagnosis of classic VS was based on the presence of at least five out of seven features considered diagnostic, as defined by Dionisi‐Vici and subsequently refined by Byrne et al.^5^, ^6^ Deep phenotyping was performed based on patient histories and brain magnetic resonance imaging (MRI) studies. Brain MRIs were assessed independently by two expert pediatric radiologists. Video‐captured movement disorder phenotypes were assessed by three movement disorders specialists who reached a consensual expert opinion. Patient data were collected using a standardized proforma including 207 items (Supplementary File 2). In addition, we reviewed all previously published patients with pathogenic *EPG5* variants and contacted lead authors for up‐to‐date information.

### Molecular Genetic Testing

*EPG5* variant screening was performed by targeted single‐gene testing or by exome/genome sequencing, as previously published.^13^ Dideoxy sequencing was performed to test for cosegregation of variants with the disorder in individual families. *EPG5* variants identified were included if classified as pathogenic or likely pathogenic according to American College of Medical Genetics and Genomics criteria.^14^ Variants of unclear significance were further interrogated applying various bioinformatic pathogenicity prediction tools.

*Epg5* ^*Q331R*^ knock‐in mice were generated by Taconic Biosciences through CRISPR/Cas9‐mediated gene editing in a C57BL/6N background (C57BL/6NTac^−^ *Epg5* ^*em4827(Q331R)Tac*^). The colony was maintained on a 12‐h light/dark cycle, and had unlimited access to food and water (PicoLab Rodent Diet 20; 5053, LabDiet, Gray Summit, MO, USA). Cage enrichment consisted of cardboard houses, tubes, and nesting materials. All procedures were carried out under the Animals (Scientific Procedures) Act of 1986 (UK) under appropriate Home Office project licenses. To characterize Epg5 levels and the autophagy defect in mice, the murine brain was divided in four separate regions (forebrain, midbrain, cerebellum, and brainstem) for RNA and protein extraction, and western blot analysis or reverse transcription quantitative polymerase chain reaction. Standardized assessments were used to characterize the behavioral phenotype of the mice at 1.5 months (6 ± 1 weeks), defined as “early stage”, and 11 ± 1 months, defined as “endstage”. As previously reported, this particular mouse model developed seizures from 10 to 12 months‐of‐age, and electrophysiological recordings documented the spontaneous occurrence of seizure‐related activity at a frequency of one per day over the course of two weeks in 10‐month‐old mice^15^. Because of the severe seizure phenotype, we have not been granted permission by the relevant UK regulatory authorities to analyze these mice beyond this stage. In line with common VS manifestations, we also assessed potential effects of Epg5 defects on hearing and muscle.

### Cellular Autophagy and Mitophagy Studies in Patient Fibroblasts and Epg5^Q331R^ Mouse Embryonic Fibroblasts

Fibroblasts carrying *EPG5* mutations homozygous for p.Gln336Arg, p.Arg1621Gln, or p.Phe2287_Leu2288insPheProThrAlaGluPhe were isolated from patient skin biopsies previously obtained as part of the routine diagnostic processes. Healthy control fibroblasts were obtained from the MRC Center for Neuromuscular Disorders Biobank, London, UK. Mouse embryonic fibroblasts (MEFs) were isolated from E15 embryos and immortalized using transformation with the SV40 plasmid according to standard protocols. Fibroblasts were either transduced with mito‐Keima (Addgene plasmid #56018) lentivirus or transfected with GFP‐Parkin (Addgene plasmid #45875) and mRFP‐GFP‐LC3 (Addgene plasmid #21074) using Human Dermal Fibroblasts Nucleofector Kit (Lonza #VPD‐1001). Cells were treated as indicated and imaged on LSM 880 Airyscan microscope using plan Apochromat ×63/1.4 oil DIC objective lens at 37°C. At least 8 z‐stacks with 0.45 μm thickness were acquired using Zen Black software (Carl Zeiss, Oberkochen, Germany). High (543/458) ratio areas and total mitochondrial area were binarized, segmented, and quantified in Fiji, and used as a mitophagy index. LC3 and GFP‐Parkin images were binarized, and puncta/cell was quantified in Fiji.

### Generation and Characterization of a Caenorhabditis elegans EPG5 Knockdown Model

Nematodes were cultured and subjected to egg‐on RNAi experiments as published.^16^ We carried out locomotion analyses^17^ in worms with knockdown in *epg‐5/EPG5*, the autophagosome‐lysosome maturation gene *rab‐7/RAB7* as a positive control for stalled late autophagy, the vacuolar fusion gene *ccz‐1/CCZ1* as a positive control for stalled fusion, and the mitophagy gene *pdr‐1/PRKN* as a positive control for a Parkinson's disorder‐related disease gene. To assess mitophagy flux, we used the strain IR621:N2;*Ex002*[p_*lgg‐1*_DsRed::LGG‐1] for measurement of the autophagosome marker LGG‐1/LC3 and the mitophagy marker DCT‐1, the ortholog of mammalian BNIP3 and BNIP3L/NIX,^18^ respectively. Three independent biological replicates of 10 hand‐picked adult day 1 nematodes each were used for microscopy. Images of nematodes with control RNAi (*luci*) and *epg‐5i* were taken with Andor Dragonfly at ×60 magnification at day 1 adulthood. Punctae counting was performed manually while analyzing researchers were blinded to image titles.

### Statistical Analysis

All data were analyzed using Prism 9 (GraphPad, San Diego, CA, USA) version 9.4.1 (458) and compiled as figures with Illustrator (Adobe, San Jose, CA, USA). Unpaired Student's *t* test was used for comparison of two groups of normally distributed data. Analyses of variances (ANOVA) with Bonferroni's multiple comparisons test was used for comparison of more than two groups.

## Results

### General Clinical Findings

We obtained data of 211 patients with pathogenic *EPG5* variants from 147 families. In addition to 97 novel cases, we obtained new follow‐up data from 88 of 114 previously published patients. There was a known history of consanguinity in 55 families. We found a family history of cancer (n = 29 patients) and neurological disorders (n = 27 patients), which included, among others, four relatives with Parkinson's disease, two relatives with dementia, four relatives with seizures, one relative with neuropathy, one relative with attention deficit disorder, and one relative with autism spectrum disorder. Supplementary File 3 shows the phenotypic data in all patients, as well as their genetic variants.

**Figure 1:**
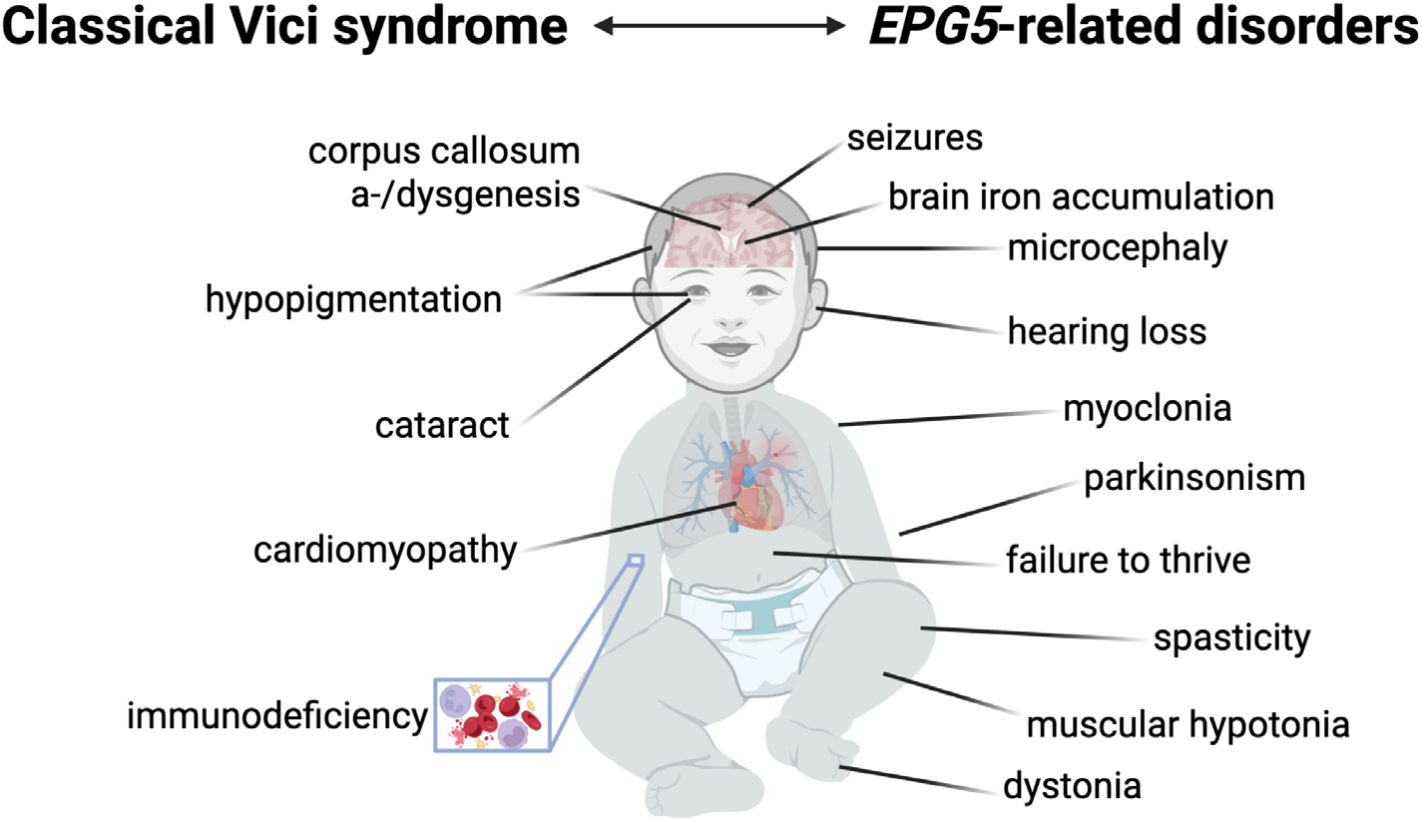
Clinical characteristics of patients within the spectrum of *EPG5*‐related disorders. Left: Features of classical Vici syndrome. Right: Spectrum of features in *EPG5*‐related disorders with the phenotypic expansion of movement disorders presented in this study, including dystonia, parkinsonism, and myoclonus. [Color figure can be viewed at www.annalsofneurology.org]

Among the key clinical diagnostic criteria for classic VS, callosal agenesis was most common (see below), but other features were more variable.^6^, ^7^ Typical clinical features for classical VS and *EPG5*‐related disorders are shown in Fig 1. The most common non‐neurological findings included failure‐to‐thrive (n = 121) and hypopigmentation (n = 107) relative to the familial and/or ethnic background. In addition to cataracts (n = 78), optic nerve atrophy (n = 50) was another common ophthalmological feature. Sensorineural hearing loss, another feature in VS,^19^ was documented in 39 cases. Cardiac involvement included mostly dilated cardiomyopathy, and a range of congenital heart defects. Microcephaly was observed in 106 cases (congenital in 18 cases and acquired in the remainder), but was less common in patients at the milder end of the spectrum. Microcephaly was often associated with other dysmorphic features (n = 79) such as micro‐/retrognathia, small bitemporal diameter, prominent upper lip, high‐arched palate, low‐set and posteriorly rotated ears, frontal bossing, and arachnodactyly.

### Genotype–Phenotype Correlation

**Figure 2:**
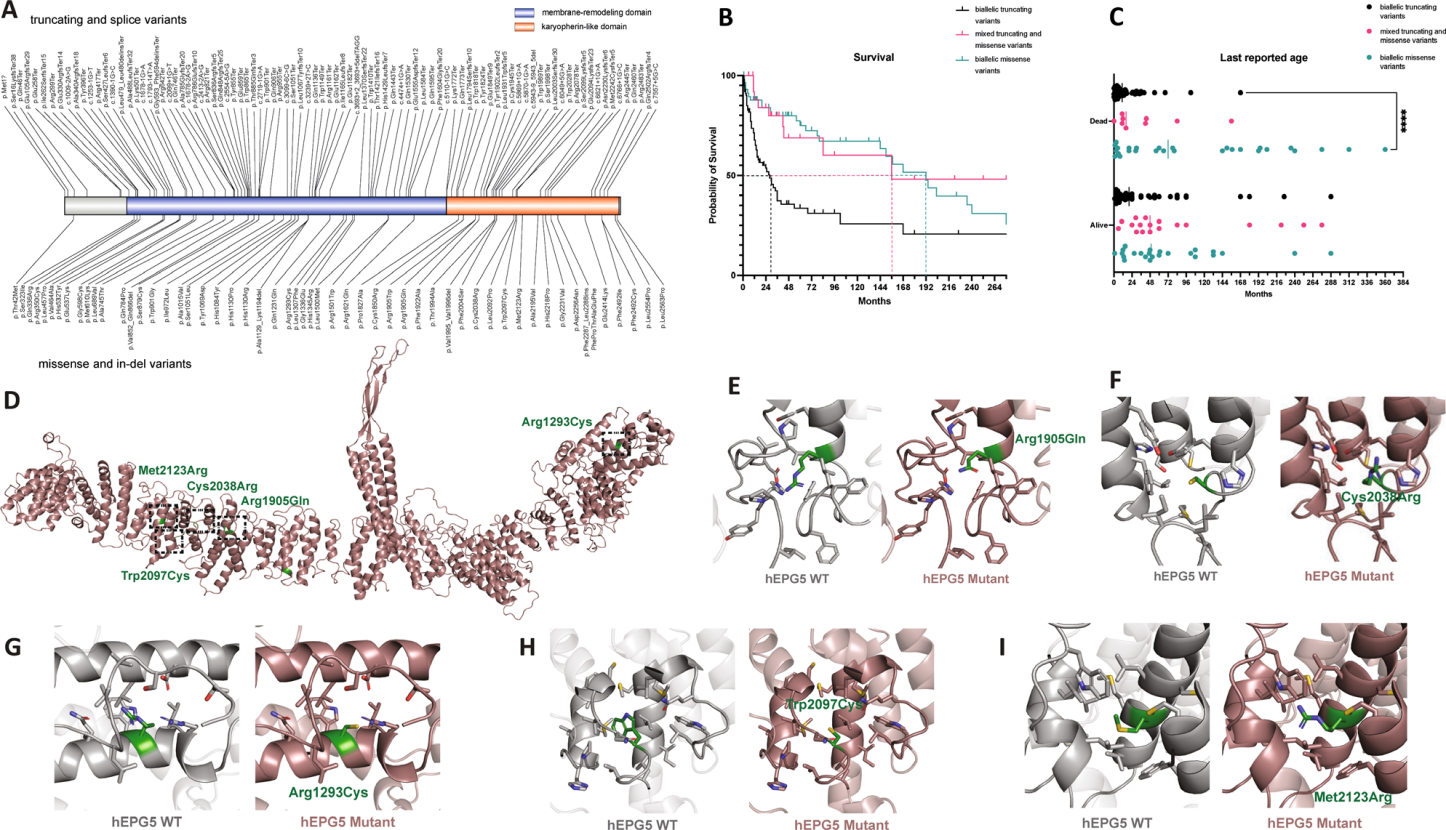
EPG5 mutational spectrum and genotype–phenotype correlations in our cohort. (A) Protein overview with selected *EPG5* variants. Truncating and splice site variants are shown above the schematic representation of the protein, missense and indel variants are pictured below (image created with IBS 2.0). (B) Kaplan–Meier survival curve of selected patients in the 3 subgroups: biallelic truncating (and splice site) variants, mixed truncating and missense variants, and biallelic missense variants. Median life expectancy noted for each curve. (C) Last reported age for each of the groups: biallelic truncating (and splice site) variants, mixed truncating and missense variants, and biallelic missense variants. (D) Overview and (E–I) specific effects of *EPG5* missense variants in relation to the EPG5 protein model, indicating changes in protein structure. More detailed descriptions in Supplementary File 1. [Color figure can be viewed at www.annalsofneurology.org]

Out of 211 patients, 123 and 88 cases carried homozygous or compound heterozygous *EPG5* variants, respectively. A total of 112 cases carried truncating and/or splice‐site variants on both alleles (“bi‐truncating”), 37 cases had a combination of one truncating/splice site variant and one missense variant (“mixed”), and 62 cases had missense variants on both alleles (“bi‐missense”). Type and distribution of *EPG5* variants identified are shown in Fig 2A. Results of in silico variant assessments are summarized in Supplementary File 4.

Where the initial diagnosis was classic VS (n = 61), *EPG5* variants were bi‐truncating in 55%, mixed in 16.5%, and bi‐missense in 28.5% of patients. Where the initial diagnosis was a non‐specific neurodevelopmental disorder (n = 150), *EPG5* variants were bi‐truncating in 22%, mixed in 13%, and bi‐missense in 65% of patients. Analysis of variance (ANOVA) confirmed a significant enhancement of bi‐truncating variants in classic VS patients. Median life expectancy also showed significant differences (*p* < 0.001, log‐rank Mantel–Cox test) between groups defined by genotype (Fig 2B,C). In particular, median life expectancy was 28 months in patients with bi‐truncating *EPG5* variants, 156 months in patients with mixed truncating and missense *EPG5* variants, and 192 months in patients with *EPG5* bi‐missense variants. Within the first month of life, 42% of patients with bi‐truncating variants are at risk of death, compared with only 15% of patients with bi‐missense variants. Protein models for the whole EPG5 protein folding and missense variants are shown in Fig 2D–I.

### Neurodevelopmental and Neuromuscular Findings

Delays of motor, speech, and/or cognitive milestones were reported in all patients. Termination of pregnancy or death in early infancy prevented the assessment of motor development in nine patients. Significant motor developmental delay was found in 189 patients surviving beyond early infancy. Epilepsy was the most common neurological feature, present in 111 and associated with a clearly progressive course in 31 patients. Eight patients were reported with generalized motor status epilepticus and non‐convulsive status. A detailed analysis of *EPG5*‐related epilepsy has been reported elsewhere.^15^

A total of 134 patients showed neuromuscular phenotypes as evidenced by clinical, muscle biopsy and/or laboratory findings. CK levels when measured were variable, but typically elevated, ranging from 50 to 2,130 IU/L.

### Neuroradiological Findings

**Figure 3:**
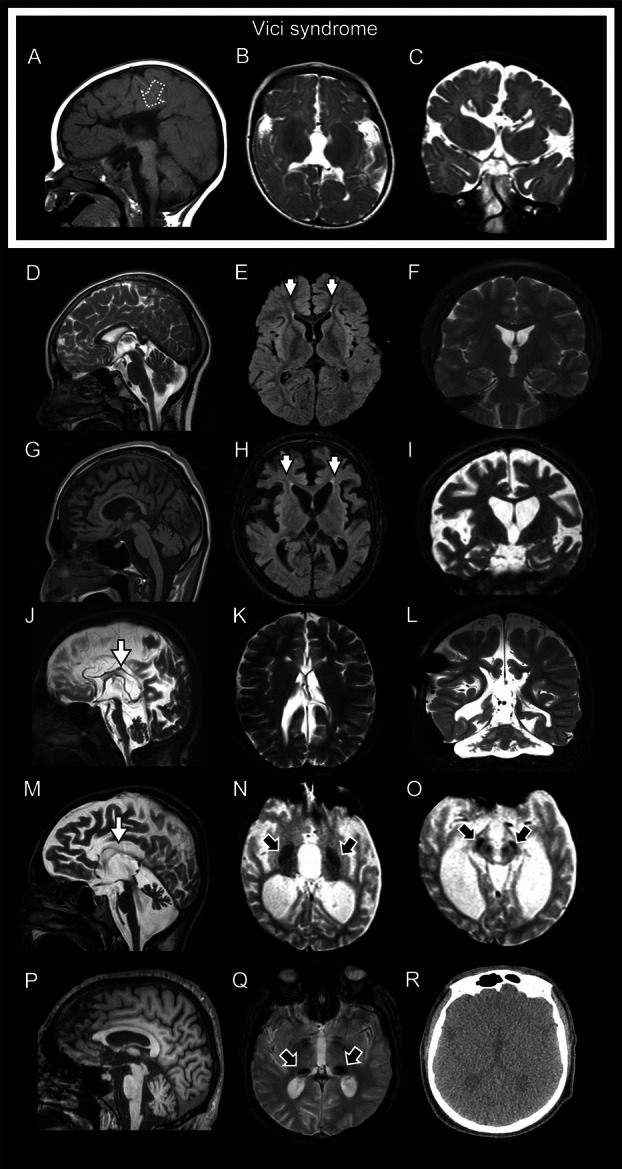
Neuroradiological spectrum in patients with *EPG5*‐related disorders. Brain magnetic resonance imaging from 6 individuals with *EPG5*‐related disorders including (A–C) one patient with Vici Syndrome and (D–R) five patients with atypical presentations. The arrow with the dotted border in (A) shows corpus callosum agenesis. Different degrees of degenerative involvement of the brain varying from mild forms with selective involvement of the fornix minors (short arrows; E,H), with or without mild corpus callosum atrophy and atrophy of the brain to more severe forms where there is an extremely thin and partially visualized corpus callosum (arrows; J–L) and cerebellar atrophy. Additional features included iron/micromineral deposition in the globus pallidi (black arrow; N), substantia nigra and red nuclei (black arrow; O), and also, as an isolated feature, in the pulvinar of the thalami (black arrows, Q).

We reviewed a total of 194 MRI and/or computed tomography scans from *EPG5*‐RD patients. Core features from representative cases are shown in Fig 3, and included structural abnormalities of the corpus callosum (mostly agenesis; n = 172), (ponto‐)cerebellar hypoplasia (n = 53), and optic nerve atrophy (n = 53). Less frequent features included schizencephaly (n = 4) and heterotopias (n = 4). Other notable additional features included thalamic involvement (n = 15), the “ear of the lynx sign” (a fluid‐attenuated inversion recovery cone shaped periventricular hyperintensity usually associated with *SPG11*‐ and *SPG15*‐spastic paraplegia; n = 3), iron accumulation in the basal ganglia (n = 5), and copper accumulation in the midbrain (n = 1; Fig 3N,O, Fig 3Q).

### An Epg5^Q331R^ Knock‐in Mouse Model Displays an Age‐Dependent Motor Phenotype

We next investigated an in vivo animal model with an *EPG5* missense variant to investigate what aspects of the neurological disorders in humans carrying recessive *EPG5* missense variants could be replicated in mice. The only published mouse model for *EPG5* defects to this date is a knockout model. The purpose for this study was to investigate whether (1) a missense mutation in mice shows a similarly mild neurodevelopmental phenotype compared to humans, and (2) if there is also an age‐dependent motor progression in mice with mild phenotypes.

The most frequent missense mutation in our human cohort is h*EPG5*:p.Gln336Arg, and is found in patients with a relatively milder phenotype than the classical Vici syndrome phenotype. For this reason, we generated a new p.Gln331Arg/Q331R knock‐in mouse model that corresponds to the human p.Gln336Arg.^20^

**Figure 4:**
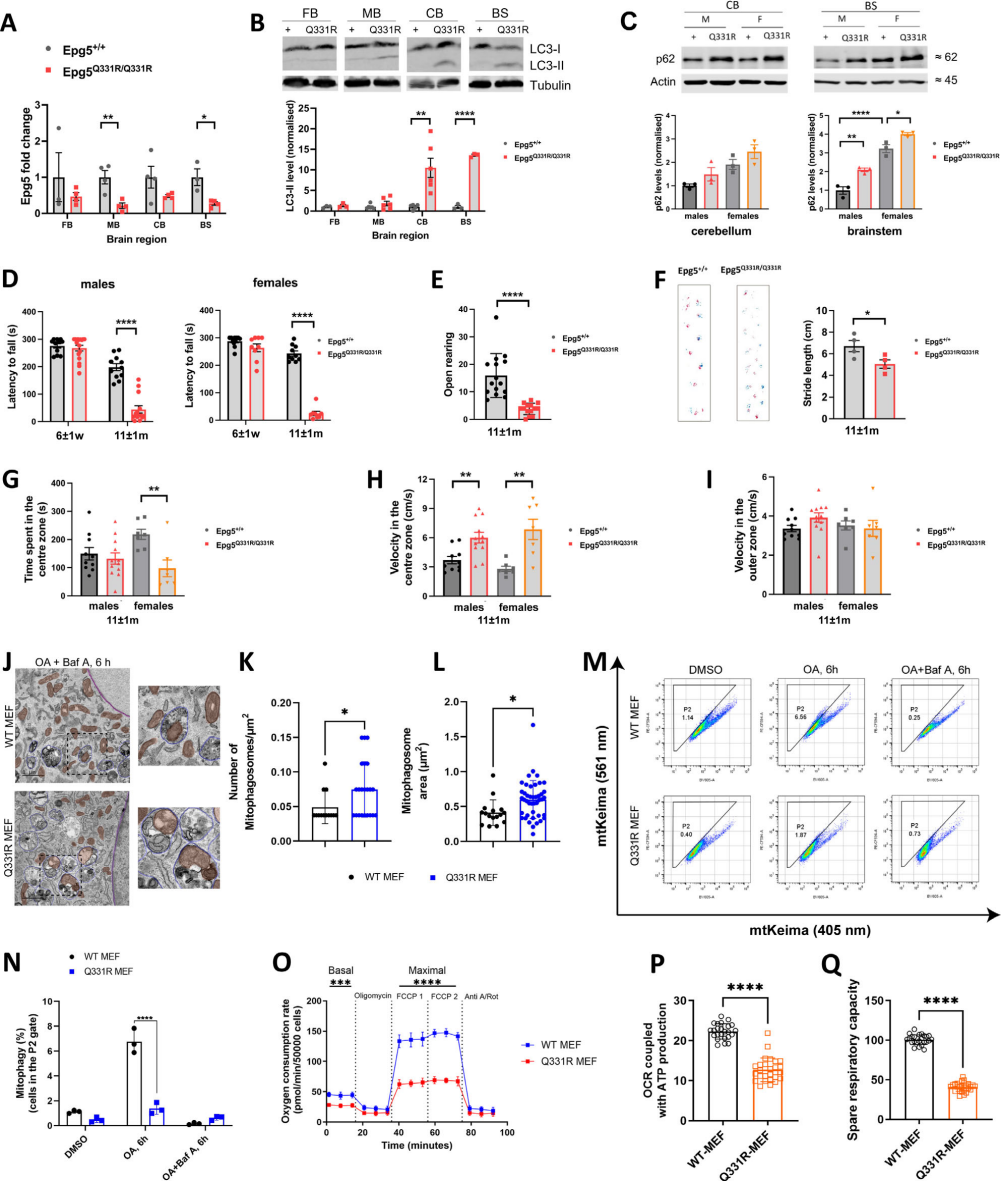
Key features from the *Epg5* ^*Q331R*^ knock‐in (KI) mouse model. (A) *Epg5* mRNA levels are significantly reduced in the midbrain and brainstem of *Epg5* ^*Q331R*^ KI mice. (B) A strong increase in LC3‐II is seen in the cerebellum and brainstem of the mutant mice. (C) A mild increase in p62 is seen in the cerebellum and a strong increase is seen in the brainstem of the mutant mice. (D) *Epg5* ^*Q331R*^ KI mice show no motor phenotype at an early stage, but fall off the rotating rod much faster than wildtype (WT) mice at endstage (~12 months). (E) At endstage, mutant mice also show reduced rearing behavior in an open arena compared with WT mice. (F) Stride length is significantly shortened in KI mice at endstage on RotaRod analysis. (G) Female *Epg5* ^*Q331R*^ KI mice spent significantly less time in the center zone of an arena than WT mice, whereas there is no significant difference between males. (H) *Epg5* ^*Q331R*^ KI mice move through the virtually indicated center zone of an arena at higher speed than WT mice, whereas (I) *Epg5* ^*Q331R*^ KI mice do not move faster through the outer zone, indicating an anxiety phenotype. (J) Representative TEM images of GFP‐Parkin expressing WT and Q331R mouse embryonic fibroblasts (MEFs) treated with oligomycin and antimycin (OA) and Baf A. Mitochondria are marked red. Scale bar, 1 μm. (K, L) Quantification of mitophagosomal number per square micron, and area in WT and Q331R MEFs stably expressing GFP‐Parkin. Plots represent the data from 3 independent experiments with n ≥ 20 TEM images. (M) FACS analysis of WT and Q331R MEFs stably expressing GFP‐Parkin and mt‐Keima. The P2 gated area encloses cells undergoing mitophagy and shows the percentage of cells within this gate of each plot. The percentages of cells within the mt‐Keima gate were much higher in wildtype cells (DMSO 1.14, OA 6 h 6.56, OA + BafA 6 h 0.25) than in EPG5 MEF cells (DMSO 0.40, OA 6 h 1.87, OA + BafA 6 h 0.73). (N) The percentage of cells undergoing mitophagy. Plots represent the data from 3 independent experiments. (O) Traces showing mitochondrial oxygen consumption rate measured in WT and Q331R MEFs using the Seahorse XFe96 extracellular flux analyzer. (P,Q) Quantitative analysis of (P) ATP‐linked respiration and (Q) spare respiratory capacity obtained from measurements in O. Data represents the mean ± SD of at least 3 independent experiments and analyzed by 1‐way ANOVA with Tukey's multiple comparisons test. *p < 0.05, **p < 0.01, ***p < 0.001, ****p < 0.0001. BS = brainstem; CB = cerebellum; FB = forebrain; MB = midbrain. [Color figure can be viewed at www.annalsofneurology.org]

Similar to humans, also in mice this variant resulted in aberrant *Epg5* mRNA splicing with retention in intron 2 (Supplementary File 5A), resulting in two isoforms with frameshifts that led to the introduction of stop codons and very low levels of correctly spliced mRNA bearing the corresponding point mutation (Supplementary File 5A,B). Consequentially, *Epg5* mRNA levels showed a significant mRNA reduction in the murine midbrain and brainstem down to less than a third, with a similar, but not statistically significant, trend in other brain areas down to half of mRNA levels when compared with wildtype (Fig 4A). In addition, autophagy defects were confirmed by LC3‐II increase in the cerebellum and brainstem, corresponding to brain areas also preferentially affected in humans, but not in the forebrain and the midbrain (Fig 4B). Corresponding to LC3‐II increases, p62 levels were also increased in those brain regions (Fig 4C), interestingly with substantially higher levels in females compared with males.

*Epg5* ^*Q331R*^ homozygous mice appeared to develop normally, and maintained a normal weight throughout early life (Supplementary File 5C). However, although the mutant mice did not show any motor phenotype on a rotarod at 6 weeks‐of‐age (Fig 4D), at ~12 months‐of‐age, there were clear deficits in latency to fall and in stride length (Fig 4D–F). A grip strength assay suggested that, in contrast to observations in *Epg5* knockout mice,^21^ the motor phenotype in the *Epg5* ^*Q331R*^ mice is likely due to reduced balance control and/or coordination rather than reduced muscle strength at this stage (Supplementary File 5D). Moreover, the absence of typical features of vestibulocochlear dysfunction (Supplementary File 5E–H) suggested that the observed balance and/or coordination issues may be a consequence of the observed cerebellar and brainstem alterations rather than of primary vestibular pathology (Supplementary File 5I,J). In conclusion, the *Epg5* ^*Q331R*^ homozygous mice showed an age‐related motor phenotype that was different from that previously reported in an *Epg5* knockout mouse model, and consistent with the expanded and milder spectrum of *EPG5*‐RDs in humans.

### Movement Disorder Phenotypes Including Early‐Onset Parkinsonism

Movement disorders were found in 80 *EPG5*‐mutated patients without primary clinical suspicion of VS, featuring spasticity (n = 55), early onset‐parkinsonism with dystonia (n = 16, detailed below), myoclonus (n = 20), or a combination of the above. In most of these patients, genetic testing (mainly through exome/genome sequencing) was only requested after the onset of the movement disorder, leading to significant diagnostic delays.

A total of 16 cases had only shown mild neurodevelopmental delay in early childhood before developing rapidly progressive “atypical parkinsonism” of sudden onset during adolescence, with additional, less prominent symptoms not typically associated with sporadic parkinsonism, including preceding dystonia, spasticity, and rapid cognitive decline. The clinical details of these 16 patients are outlined below.

A 14‐year‐old boy (patient 79.1), compound heterozygous for *EPG5* variants p.Gly2231Val and c.4474+1G>A, presented in adolescence on the background of moderate neurodevelopmental delay with a complex movement disorder of subacute onset, including prominent parkinsonism, generalized dystonia with orofacial dyskinesia, and intermittent jerky action tremor. He progressed to almost complete tetraparesis within two years of disease onset. Brain MRI findings included iron or mineral accumulation in the pulvinar (Fig 3V). Positron emission tomography computed tomography showed pronounced hypometabolism in the parietal and right occipital lobes, and in the posterior cingulate gyrus, with severe synaptic dysfunction in these brain regions. Similar, but less pronounced, abnormalities were also found on the left side. There was additional mild, but definite, upregulation of striatal metabolism (Supplementary File 6). Dopamine transporter scan showed pronounced degeneration in presynaptic dopamine transporters as a consequence of pronounced nigrostriatal degeneration of the dopaminergic system, equally affecting the caudate nucleus and putamen.

A 14‐year‐old girl (case 96.1) compound heterozygous for *EPG5* variants p.Val464Ala and p.Tyr855Ter presented with severe progressive fluctuating generalized dystonia, parkinsonism, and cognitive decline on the background of moderate neurodevelopmental delay. Brain MRI studies demonstrated callosal agenesis, generalized atrophy, with additional features suggestive of basal ganglia iron accumulation in the substantia nigra and red nuclei (Fig 3S,T).

A 14‐year‐old girl (case 114.1) compound heterozygous for *EPG5* variants p.Arg1501Trp and p.Thr1994Ala presented with generalized dystonia and parkinsonism with cognitive decline followed by rapid deterioration and death at the age of 16 years. She had a background of global developmental delay, congenital hydrocephalus, and progressive optic nerve atrophy. Brain MRI studies showed callosal dysgenesis, pontocerebellar hypoplasia, and thalamic involvement (Fig 3O–Q).

An 18‐year‐old woman from a consanguineous family (case 134.1) homozygous for the *EPG5* variant c.5943‐9_5943‐5del presented with parkinsonism with anarthria, an extraocular movement disorder, generalized dystonia pronounced in the oropharyngeal muscles, spasticity, an irregular jerky tremor in the lower limbs, and cognitive decline. She had a background of global developmental delay, optic nerve atrophy, and complex partial seizures responsive to levetiracetam from the age of three years. Brain MRI studies performed at the age of 18 years showed generalized atrophy. She had a rapidly progressive course and died at the age of 18 years.

Two siblings homozygous for the *EPG5* variant p.Phe2004Ser had a background of global severe neurodevelopmental delay. The older brother (136.1) developed dystonia, choreoathetosis, spasticity, dysarthria, parkinsonism with tremor, postural instability, and hypomimia in adolescence, followed by rapid cognitive decline with sleep disturbance, anxiety, and death at the age of 20 years. MRI of the brain showed cerebral and cerebellar atrophy, callosal agenesis, lynx sign, and pallidal T2‐hyperintensities on MRI (Fig 3J). The younger sister (136.2) presented from 12 years with dystonia and parkinsonism followed by slow regression on a background of intellectual disability. She subsequently developed seizures responsive to phenytoin from 19 years, but died after further disease progression. MRI of the brain was consistent with periventricular leukodystrophy.

Two sibling pairs (Patients 137.1–137.4) who were first cousins from a large consanguineous family were homozygous for the *EPG5* variant p.Phe2287_Leu2288insPheProThrAlaGluPhe. All four patients presented with variable combinations of parkinsonism, dystonia, tremor, and spasticity from their teenage years on a background of global developmental delay and mild intellectual disability. MRI showed callosal dysgenesis, lynx sign, generalized atrophy, and ventriculomegaly (137.2 in Fig 3M). The disease course was progressive with cognitive decline in all, and premature death at the age of 26 years following further deterioration in one case (patient 137.3).

Patient 143.1 was homozygous for the *EPG5* variant p.Ser547Phe, and showed congenital microcephaly, spasticity, nystagmus at birth, and seizures from infancy on the background of a global neurodevelopmental disorder with moderate to severe intellectual disability. He developed parkinsonism from the age of nine years. Brain MRI studies showed heterotopia, but no other abnormalities.

Two siblings from a consanguineous family (145.1 and 145.2) were homozygous for the *EPG5* variant p.Pro1309Leu, and presented with a global neurodevelopmental disorder, hypopigmentation, seizures, spasticity, failure to thrive, and microcephaly. Brain MRI studies showed corpus callosum dysgenesis and pontocerebellar hypoplasia, as well as optic nerve atrophy in both patients. The elder sister (145.1) developed parkinsonism from the age of 19 years and the younger brother (145.2) showed parkinsonism from the age of 11 years. Both patients also showed paroxysmal chorea, joint and finger contractures, scoliosis, and hypertrichosis.

Patient 147.1 was compound heterozygous for the *EPG5* variants p.Val1238Ala and p.Trp2420Cys. He initially presented with mild to moderate intellectual disability (IQ 45–51) and autism. At the age of 17 years, he developed a rapidly progressive choreiform movement disorder with dystonia in the left hand and spasticity, eventually showing parkinsonism with bilateral bradykinesia, rigidity, and resting tremor that was treated with levodopa/carbidopa to a limited effect. Brain MRI showed cerebellar atrophy, a bilateral symmetrical T2, and SWI hypointensities in the pulvinar reminiscent of iron deposition in Parkinson's disease patients.

In addition, two particularly severely affected siblings (90.1 and 90.2) compound heterozygous for *EPG5* variants c.5869+1G>A and p.Trp1989Ter presented with myoclonus from birth, followed by dystonia from four weeks, early‐onset parkinsonism at three months, progressive infantile epileptic encephalopathy, and death in late infancy.

### EPG5 Patient Fibroblasts Show Impaired PINK/Parkin‐Dependent Mitophagy with Defective Removal of Stressed Mitochondria

To detail at cellular level the effect of *EPG5* mutations involved in the expanding spectrum of *EPG5*‐related movement disorders including parkinsonism and distinct from classical VS, we investigated human fibroblasts homozygous for the *EPG5* variant c.6861_6862insTTTCCAACAGCAGAGTTC, p.Phe2287_Leu2288insPheProThrAlaGluPhe identified in *EPG5*‐related parkinsonism (patient 3), as well as two fibroblast cell lines with the recurrent *EPG5* Vici syndrome variants p.Gln336Arg and p.Arg1621Gln (patients 1 and 2), respectively. The cell line from the *EPG5*‐mutated patient with parkinsonism was chosen to investigate for clear mitophagy defects as a cause for potential neurodegeneration. The two VS‐related *EPG5* variants were chosen to investigate if cell lines from patients with earlier and more severe clinical manifestations may also show mitophagy defects, which may in turn prompt neurodegeneration at an age where thalamic motor networks are not yet fully developed.

**Figure 5:**
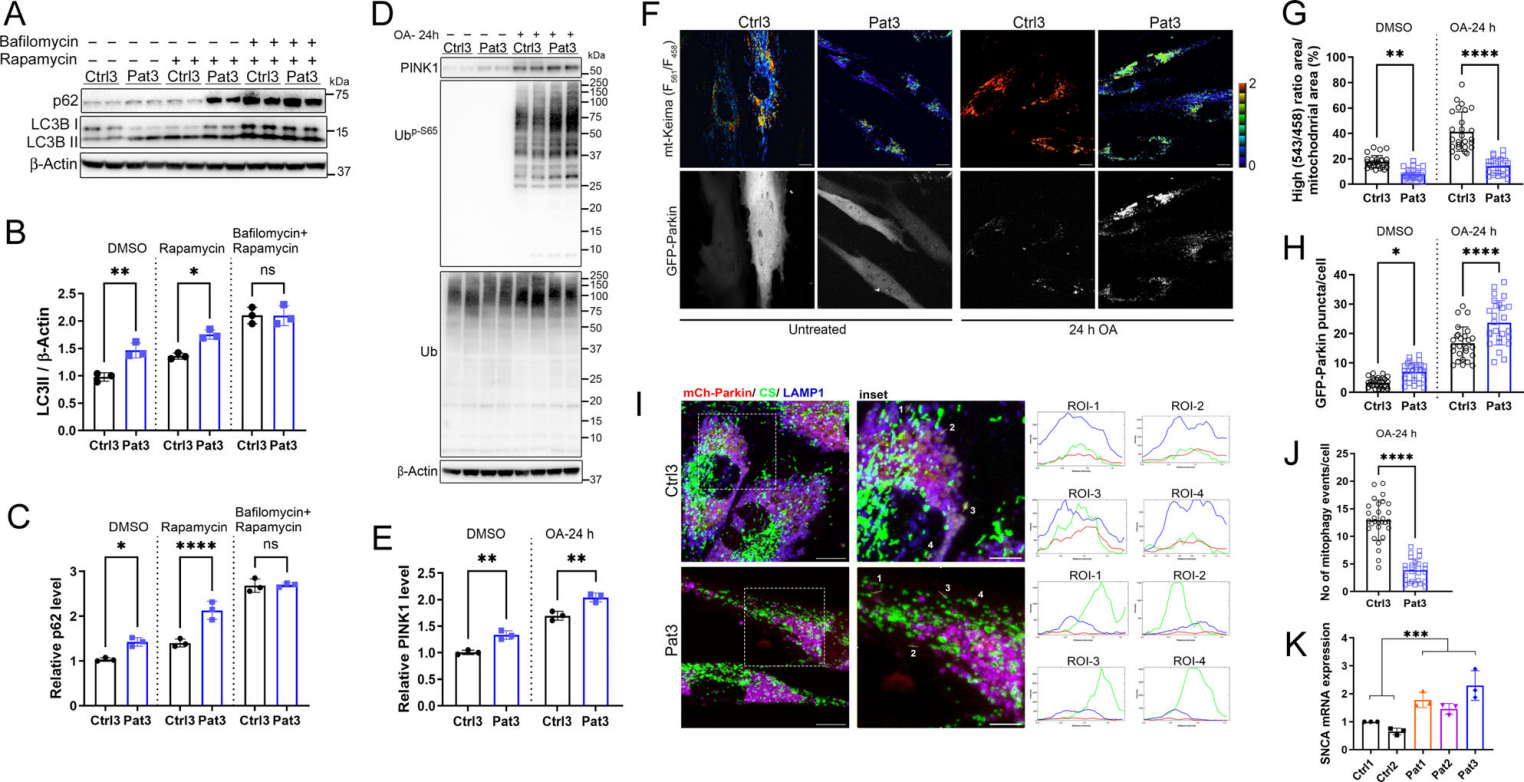
Impaired autophagic flux and mitophagy in cells from a patient with *EPG5*‐related parkinsonism and *SNCA* overexpression. (A) Utilizing a fibroblast cell line from a 22‐year‐old patient with a homozygous *EPG5* variant in c.6861_6862insTTTCCAACAGCAGAGTTC, p.Phe2287_Leu2288insPheProThrAlaGluPhe, we conducted immunoblots showing accumulation of p62 and LC3II in control and patient fibroblasts after 24 h of treatment with rapamycin (100 nM) and/or bafilomycin (200 nM). (B, C) Quantification of LC3II/β‐Actin ratio and p62 levels in A. (D) Immunoblot showing an increase of of PINK1 levels and its activity (phospho‐Ub) in control and patient fibroblasts in response to oligomycin (2.5 μM) and antimycin (2.0 μM; OA) treatment for indicated time. Ubiquitin levels (Ub) blot shows the total ubiquitin levels. (E) Quantification of the PINK1 levels in D. (F) Representative images of fibroblast cells cotransfected with mito‐Keima (ratiometric) and GFP‐Parkin (gray scale) before and after the treatment with OA for 24 h. Scale bars, 10 μm. (G) The proportion of the high ratio (561/458) signal area (red) to the total mitochondrial area plotted as mitophagy index. (H) Quantification of the number of GFP‐positive puncta per cell. Each experiment examined at least 12 transfected cells. (I) Representative immunofluorescence images of mCherry‐Parkin transfected fibroblasts immunostained for citrate synthase (CS) and LAMP1. Scale bar: overview, 10 μm; inset, 5 μm. Regions of interest (ROI) show line profile of Parkin‐CS puncta with the LAMP1‐positive vesicles, completely colocalizing in control fibroblasts. These structures were counted as “mitophagy events”, which were further plotted. (J) Quantitative analysis of the number of mitophagy events in H. Data in B, C, E, G, I, J, and K are represented as mean ± SD from 3 independent experiments. (K) Quantitative analysis of *SNCA* transcript in control and patient fibroblasts shows overexpression in cells from patients with the spectrum of *EPG5*‐related neurodevelopmental disorders (“attenuated VS spectrum”) in patient 1 (Q336R) and patient 2 (R1621Q) from Figure 6, as well as in the cell line from the patient 3 with *EPG5*‐related parkinsonism. *p < 0.05, **p < 0.005, ***p < 0.001, ****p < 0.0001 (2‐tailed unpaired Student *t* test or 1‐way ANOVA); ns = Not significant. [Color figure can be viewed at www.annalsofneurology.org]

Monogenic disorders of mitophagy with PINK1 or Parkin/PRKN deficiency are associated with early‐onset parkinsonism in humans. We next investigated if any PINK1/Parkin‐mediated mitophagy defect at cellular level may be associated with the parkinsonism that we observed in our patients. More specifically, we investigated a fibroblast cell line from a 22‐year‐old patient with parkinsonism and a homozygous *EPG5* variant in c.6861_6862insTTTCCAACAGCAGAGTTC, p.Phe2287_Leu2288insPheProThrAlaGluPhe. Immunoblots showed accumulation of LC3II and p62 in patient fibroblasts following vehicle and rapamycin treatments (Fig 5A–C). We observed accumulation of PINK1 levels and its activity (phospho‐Ub) in the patient cell line in response to mitochondrial damage with 24‐h oligomycin‐antimycin treatment (Fig 5D,E).

After cotransfecting this cell line with mito‐Keima and GFP‐Parkin, we observed significant downregulation of mtKeima high ratio in patient cells already at vehicle treatment, as well as after OA‐induced mitochondrial damage, indicating stalled mitophagy (Fig 5F,G). In addition, we observed a significant upregulation in GFP‐Parkin punctae, even with vehicle treatment and more so following OA‐induced mitochondrial damage (Fig 5F,H). Analyses of immunofluorescence studies in mCherry‐Parkin transfected fibroblasts immunostained for citrate synthase (CS) and LAMP1 showed Parkin‐CS punctae mostly colocalizing with LAMP1‐positive vesicles in control fibroblasts, but this colocalization was significantly decreased in patient cells, indicating a reduced number of mitophagy events (Fig 5I,J).

Finally, we examined α‐synuclein overexpression, a specific biomarker for parkinsonism with PINK1 defects and leading to dopaminergic loss, by quantifying mRNA abundance for its gene *SNCA*, as documented in several previous studies.^22^, ^23^ We observed α‐synuclein overexpression through a significant upregulation of *SNCA* mRNA abundance in cells from patients at the relatively milder end of the *EPG5*‐related VS spectrum in patient 1 (p.Gln336Arg) and patient 2 (p.Arg1621Gln), as well as in the cell line from patient 3 with *EPG5*‐related parkinsonism (Fig 5K).

Next, we investigated whether the same findings of defective PINK1‐PRKN‐mediated mitophagy in the fibroblast cell line from a patient with *EPG5*‐parkinsonism are found in fibroblast cell lines from patients with milder *EPG5*‐related disorders at earlier ages and without parkinsonism at time of biopsy (p.Gln336Arg and p.Arg1621Gln). Both variants are recurrent founder variants.

**Figure 6:**
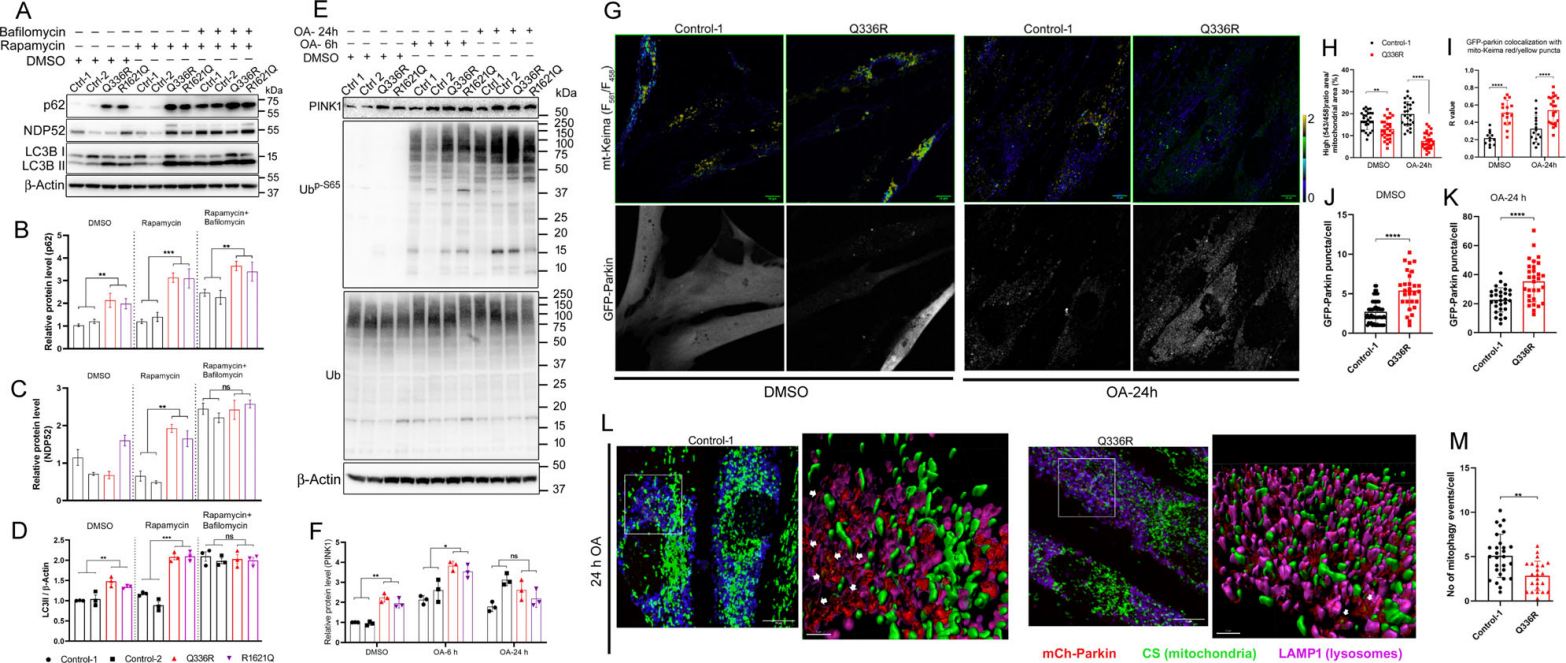
Impaired autophagic flux and mitophagy defects in *EPG5*‐mutated patient cells. (A) Immunoblot showing accumulation of p62, NDP52, and LC3II in control and patient fibroblasts after 24 h of treatment with rapamycin (100 nM) and/or bafilomycin (200 nM). Quantifications of (B) p62 levels in, (C) NDP52 levels, and (D) LC3II/β‐Actin ratio, respectively. (E) Immunoblot showing increase of PINK1 levels and its activity (phospho‐UbS65) in relation to total ubiquitin levels (Ub) in both Q336R and R1621Q fibroblasts in response to oligomycin (2.5 μM) and antimycin (2.0 μM; OA) treatment for indicated time, (F) with quantification of PINK1 levels. (G) Representative images of fibroblast control and Q336R patient cells cotransfected with mito‐Keima (ratiometric) and GFP‐Parkin (gray scale) under vehicle treatment with DMSO (left) and after treatment with OA for 24 h (right). Scale bars, 10 μm. (H) Proportion of the high ratio (561/458) signal area (red) to the total mitochondrial area plotted as mitophagy index. (I) Pearson coefficient indices between GFP‐Parkin and mito‐Keima (high ratio area) show the accumulation of GFP‐Parkin on the mitophagosomes/mitolysosomes over time under normal and OA‐treated condition. (J, K) Quantification of the number of GFP‐positive puncta per cell in DMSO and OA 24 h treatment, respectively. Each experiment examined n ≥ 20 transfected cells. Scale bar: 10 μm. (L) Representative immunofluorescence images of mCherry‐Parkin transfected fibroblasts immunostained for citrate synthase (CS) and LAMP1. 3D reconstruction of inset in Control‐1 shows Parkin‐CS puncta inside the LAMP1‐positive vesicles (white arrows). These structures were counted as “mitophagy events” and quantitatively demonstrated in (M). Scale bar: overview, 10 μm; inset, 2 μm. Data in (B–D and F) are represented as mean ± SD from 3 independent experiments. *p < 0.05, **p < 0.005, ***p < 0.001, ****p < 0.0001 (2‐way ANOVA). ns = Not significant. [Color figure can be viewed at www.annalsofneurology.org]

We confirmed the expected autophagy blockade in these cells and demonstrated, compared with controls, higher levels of autophagy flux markers p62/SQSTM1, NDP52, and LC3‐II on immunoblotting, already under basal condition, and increased even further following treatment with rapamycin and bafilomycin (Fig 6A–C). EPG5‐defective fibroblasts transfected with tandem‐fluorescent mRFP‐GFP‐LC3 demonstrated autophagosome accumulation before fusion with lysosomes (Supplementary File 7A,B).

During mitophagy induction, we observed colocalization of EPG5 and fragmented mitochondria in response to mitochondrial depolarization induced by oligomycin and antimycin in control fibroblast cell lines (Supplementary File 7C). To demonstrate early mitophagy inhibition, PINK1 levels were already significantly elevated in patient cells at baseline, and increased even further after 6‐h treatment with oligomycin and antimycin (Fig 6E,F). We observed a time‐dependent increase in levels of ubiquitin phosphorylation at Ser65 upon oligomycin‐antimycin treatment, which showed a stall in the PINK1‐mediated post‐translational modification essential for mitochondrial recruitment during mitophagy.^24^ We observed PINK1 activation on depolarized mitochondria in immunostaining for Ub^p‐S65^, TOM20, and LAMP1 in patient cells (Supplementary File 7D,E). Quantification of Ub^p‐S65^ signal on mitochondria indicated robust PINK1 activation (Fig 6F, Supplementary File 7D). Colocalization analysis of Ubp^S65^ with lysosomes showed a significant decrease in p.Gln336Arg cells (Supplementary File 7F), indicating defective delivery of phospho‐ubiquitinated mitochondria to lysosomes.

We investigated whether Parkin is recruited to damaged mitochondria during oligomycin‐antimycin‐induced mitophagy in patient cells. Delivery of mt‐Keima to lysosomes at 24‐h oligomycin‐antimycin treatment resulted in the formation of bright punctae with a high ratio of excitation at 543/458 nm in response to the acidic lysosomal microenvironment in control cells,^25^ and this was significantly reduced in patient cells showing reduced mitophagy flux (Fig 6G,H). With vehicle treatment, GFP‐Parkin was localized diffusely throughout control fibroblasts, whereas p.Gln336Arg fibroblasts showed a significant increase in GFP‐punctae colocalizing with low F_543_/F_458_ mt‐Keima signal (Fig 6I,J). GFP‐Parkin recruitment was further increased in p.Gln336Arg cells after 24‐h oligomycin‐antimycin treatment during late mitophagy (Fig 6K). A lower GFP‐Parkin signal colocalizing with high F_543_/F_458_ mt‐Keima signal in wildtype indicated degradation of the fusion protein in mitolysosomes, whereas patient cells showed significantly higher GFP‐Parkin and high mtKeima signal colocalization indicating stalled Parkin‐mediated mitophagy (Fig 6G–K). Fig 6L shows the delivery of Parkin‐positive mitochondria to lysosomes in cells transfected with mCherry‐Parkin, followed by oligomycin‐antimycin treatment and immunostaining for mitochondrial matrix protein, CS, and LAMP1. Quantification of Parkin‐positive CS punctae colocalizing with lysosomes, identified as mitophagy events, were significantly decreased in p.Gln336Arg cells, indicating defective degradation and accumulation of Parkin‐activated mitochondria (Fig 6M). These findings indicated that the autophagosome–lysosome fusion defect in EPG5 deficiency also extends to the removal of stressed mitochondria in fibroblast cell lines from patients with milder *EGP5*‐related disorders (p.Gln336Arg and p.Arg1621Gln).

As our knock‐in mice carried the genetic alteration at a homologous amino acid residue as humans with the recurring founder variant p.Gln336Arg, we next investigated on a cellular basis whether immortalized MEFs from *Epg5* ^*Q331R*^ homozygous mice show similar defects in PINK1/Parkin‐dependent mitophagy, as evidenced in human fibroblasts above. We used transmission electron microscopy to identify the downstream block in mitophagy by analyzing mitophagosomal structures in MEFs stably expressing GFP‐Parkin (Fig 4J). Consistent with increased accumulation of mitophagosomes in patient fibroblasts, *Epg5* ^*Q331R*^ MEFs showed a significant increase in mitophagosomes during PINK1/Parkin mitophagy in the presence of BafA, compared with wildtype (Fig 4K). Notably, mitophagosomes formed in *Epg5* ^*Q331R*^ MEFs contained correctly sequestered mitochondria, although those were significantly larger than in wildtype cell lines (Fig 4L), further supporting impaired lysosomal fusion of normally formed lysosomes as a key mechanism. In MEFs stably expressing GFP‐Parkin and mt‐Keima, quantitative mitophagy measurements by FACS analysis showed a significant increase in mt‐Keima spectral shift upon OA‐induced mitophagy in wildtype, which was reversed by cotreatment with BafA (Fig 4M). *Epg5* ^*Q331R*^ homozygous MEFs also had a largely attenuated mt‐Keima spectral shift in response to OA (Fig 4N), again indicating lower levels of PINK1/Parkin‐mitophagy. Finally, we analyzed mitochondrial oxygen consumption rate in Q331R MEFs and wildtype MEFs using the Seahorse XFe96 extracellular flux analyzer (Fig 4O), and observed a significant reduction in Q331R MEFs in both ATP‐linked respiration (Fig 4P) and spare respiratory capacity (Fig 4Q).

Taken together, cellular data from MEF derived from mice with EPG5 defects confirm the causal relationship between EPG5 recessive variants and mitochondria abnormalities.

In conclusion, defective mitophagy and accumulation of dysfunctional mitochondria appears to be a common element at a cellular level throughout the *EPG5*‐RD continuum.

### epg‐5 Knockdown in C. elegans Causes Impaired Mitophagy

After having established the association of defective removal of stressed mitochondria in mice and human cells in vitro, as well as age‐dependent motor abnormalities in mice in vivo, we investigated whether these observations extend to other recognized autophagosome–lysosome fusion defects and/or those already associated with Parkinson's disease.

**Figure 7:**
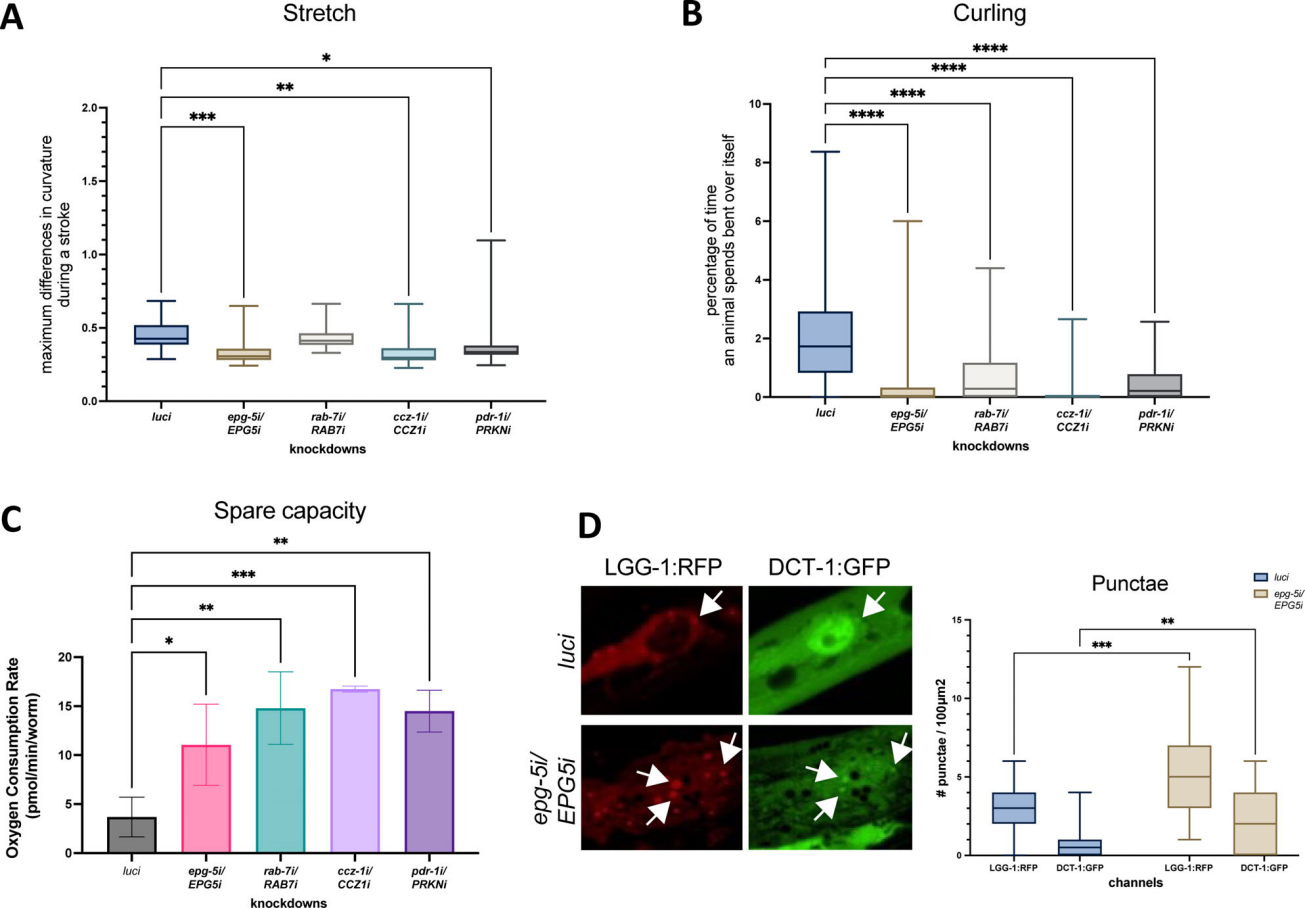
*Caenorhabditis elegans* model of *epg‐5/EPG5* dysfunction. Motor phenotype, mitochondrial oxygen consumption, and mitophagy flux in *Caenorhabditis elegans* knockdown of *epg‐5/EPG5*. (A) Analysis of stretch showed significantly decreased maximum differences in curvature per stroke in *epg‐5i/EPG5i*, *ccz‐1i/CCZ1i*, and *pdr‐1i/PRKNi*, indicating flatter body bends, demonstrated by pooled data from 3 biological replicates of n = 10 worms. (B) Analysis of curling showed significantly decreased percentage of time spent in bent‐over shapes in *epg‐5i/EPG5i*, *rab‐7i/RAB7i*, *ccz‐1i/CCZ1i*, and *pdr‐1i/PRKNi* worms, indicating much flatter movements, demonstrated by pooled data from 3 biological replicates of n = 10 worms. (C) Measurement of oxygen consumption rate by Seahorse Respirometer revealed an increase in spare capacity in *epg‐5i/EPG5i*, *rab‐7i/RAB7i*, *ccz‐1i/CCZ1i*, and *pdr‐1i/PRKNi*, indicating an excess of uncoupled capacity of the respiratory electron transport chain not being used in basal respiration. (D) Microscopy of mitophagy flux markers revealed an increase in LGG‐1:RFP and DCT‐1:GFP puncta in body wall muscle cells when compared with *luci* control. Quantification of punctae per 100 μm^2^ visual counting, shown as pooled data from 3 biological replicates of n = 10 worms. Statistical significance levels: *p ≤ 0.05, **p ≤ 0.01, ***p ≤ 0.001 as determined by ANOVA. [Color figure can be viewed at www.annalsofneurology.org]

As an easily genetically manipulated in vivo model for motor phenotypes and mitochondrial function, we conducted knockdown of *epg‐5/EPG5* in a *C. elegans* model, and observed a hypokinetic phenotype with decreased body bends and lower thrashing similar to knockdown of both other autophagosome–lysosome fusion genes (*rab‐7/RAB7A, ccz‐1/CCZ1*) and of Parkinson's disease‐associated gene *pdr‐1/PRKN* (Fig 7A,B), corroborating previous findings in established *C. elegans* models of Parkinson's disease.^26^ These findings showed that the observed motor phenotypes are not selective to *epg‐5/EPG5* dysfunction, but rather a consequence of any autophagosome–lysosome fusion and/or mitophagy defect.

To further investigate for specific aspects of mitochondrial dysfunction, we conducted SeaHorse analyses in *C. elegans* knockdown models. Compared with control *luci* worms, oxygen consumption assays in *epg‐5*‐deficient *C. elegans* showed an increase in the differential between maximum and basal oxygen consumption rate (“spare capacity”), indicative of an excess of uncoupled capacity of the respiratory electron transport chain, similar to observations with knockdown of other autophagosome–lysosome fusion (*ccz‐1/CCZ1*) and Parkinson's disease‐associated genes (*pdr‐1/PRKN*) in worms (Fig 7C). Again, these findings suggested that the observed mitochondrial dysfunction is not selective to *epg‐5/EPG5* defects, but rather a consequence of any autophagosome–lysosome fusion or mitophagy defect.

Finally, to investigate for stalled mitophagy flux, we examined *epg‐5/EPG5* knockdown in a mitophagy reporter strain that showed increased punctae of LGG‐1:RFP (homolog of autophagosome marker LC3) and DCT‐1:GFP (homolog of mitophagy marker BNIP3L/NIX) in body wall muscles from *epg‐5/EPG5* knockdown worms when compared with control (Fig 7D). This finding showed that the dysfunctional removal of stressed mitochondria is not only found in human and mouse fibroblast cells, but also evident in vivo in worms.

In summary, findings in *C. elegans* models correspond to motor abnormalities and mitophagy flux dysregulation seen in *EPG5*‐mutated mice and humans. More specifically, worm assays show that *EPG5*‐related autophagolysosome fusion defects are downstream of defects in formation of mito‐autophagosomes in vivo. In conclusion, defective mitochondria and motor abnormalities are the consequence of any autophagosome–lysosome fusion defect (CCZ1, RAB7A, EPG5).

## Discussion

Here, we present genetic, clinical, and imaging data from the largest cohort of patients with pathogenic *EPG5* variants reported to date. We provide evidence for an expanding phenotypic range associated with EPG5 defects, suggesting a relatively milder end of the clinical spectrum different from classic VS,^27^ and a continuum with other neurodegenerative phenotypes of later onset. Although classic VS may be rare, our findings suggest that other *EPG5*‐RDs may be more common, including more frequent, but less specific, presentations, such as neurodevelopmental delay, epilepsy, spasticity, dystonia, and early‐onset parkinsonism.

Not unexpectedly, we found patients with classic VS, typically identified through *EPG5* Sanger sequencing prompted by suggestive clinical features, with at least 1 truncating *EPG5* variant allele, expected to result in reduced EPG5 protein expression, whereas those with less specific clinical diagnoses before genetic testing were typically identified through an unbiased exome/genome sequencing approach and more often found with at least one missense variant allele, expected to result in dysfunctional EPG5 protein. This may suggest a dosage effect in *EPG5*‐RD, with the phenotype of classic VS associated with marked reduction of the EPG5 protein and relatively milder phenotypes associated with expression of a dysfunctional EPG5 protein. However, further follow‐up studies are required to establish the hypothesized dosage effect in more detail. A dosage effect is also suggested by the previous preliminary observation of movement disorders in heterozygous *EPG5* variant carriers in a small number of families, and the suggestion of *EPG5* variants as a modifying factor in Alzheimer's disease and amyotrophic lateral sclerosis.^7^, ^28^, ^29^ Interestingly, an *epg5*‐related dosage effect was also seen in a *Drosophila* model where heterozygous knockout and knockdown flies showed a milder phenotype compared with homozygous knockouts.^15^

We identified specific novel clinical and radiological presentations within the spectrum of *EPG5*‐RDs that mimicked a number of monogenic disorders characterized by dystonia, hereditary spastic paraplegia, and early‐onset parkinsonism. This expanded *EPG5*‐RD cohort suggested prominence of motor disorders with either: (1) already postnatal or infantile onset, or, more commonly, (2) adolescent‐onset dystonia and parkinsonism, typically on the background of non‐specific neurodevelopmental delay. Early‐onset parkinsonism as part of *EPG5*‐RDs corresponds to earlier preliminary observations of an apparently increased Parkinson's disease risk in heterozygous *EPG5* variant carriers. The iron accumulation in the basal ganglia seen in some *EPG5*‐mutated patients has previously been reported as part of the MRI spectrum in *PKAN*‐ or *WDR45*‐associated neurodegeneration with brain iron accumulation.^30^ Early radiological features in *WDR45*‐mutated patients may closely mimic those of VS, supporting a clinico‐radiological continuum between those disorders. Albeit novel, the observed overlap of EPG5 defects with disorders of iron metabolism is not unexpected, considering the crucial role of autophagy in iron homeostasis.^31^

As a notable finding in this study, we investigated a fibroblast cell line from a patient with *EPG5*‐related parkinsonism that showed defects in PINK1/Parkin‐mediated mitophagy, reiterating our findings in cell lines from milder *EPG5*‐related disorders. Our cellular studies in human and murine fibroblasts carrying recurrent homozygous *EPG5* missense variants with a milder phenotype also suggest mitochondrial dysfunction, as well as defects in PINK1/Parkin‐mediated mitophagy. Although mitochondrial dysfunction may happen at any time in cells, the clearance of dysfunctional mitochondria requires a steady state of mitophagic flux. Our findings suggest that mitophagic flux is severely impaired in EPG5 deficiency, as EPG5 controls the bottleneck of general autophagolysosome tethering, which is downstream of the formation of mito‐autophagosomes.

Monogenic defects in mitophagy, such as PINK1 or Parkin deficiency, are associated with early‐onset parkinsonism, and there is a significant phenotypic overlap with our *EPG5*‐mutated patients with parkinsonism.

Our *EPG5*‐related findings correspond to observations in *PARK2*‐ and *PINK1*‐mutated patient cells and induced dopaminergic neurons, showing accumulation of damaged mitochondria upon induction of mitophagy.^32^ Histochemical analysis of substantia nigra sections from *PARK2*‐ and *PINK1*‐mutated patients also feature pS65‐Ub punctae colocalizing with mitochondria and lysosomes similar our observations in *EPG5*‐mutated cells.^33^ The impaired mitophagy observed in *EPG5*‐mutated patient cells also closely mimics mitochondrial abnormalities seen in a mouse model of *GBA*‐associated Gaucher disease, a lysosomal storage disorder associated with Parkinson's disease in heterozygous *GBA* variant carriers.^34^ One of the most prominent biomarkers in monogenic disorders with parkinsonism is α‐synuclein, a protein that is accumulated in Lewy bodies of dopaminergic neurons from patients with parkinsonism as a molecular correlate for neurodegeneration; several studies have shown α‐synuclein accumulation in a number of monogenic disorders relevant to this study, such as in *GBA*‐related parkinsonism.^35^ Notably, we were able to show α‐synuclein overexpression through *SNCA* mRNA abundance in the cell line from the patient with *EPG5*‐related parkinsonism, as well as in the 2 cell lines from *EPG5*‐mutated patients with milder, primarily neurodevelopmental disorders. This finding shows the role of EPG5‐mediated autophagolysosome fusion in adequate mitophagic flux, that when perturbed may eventually lead to clinical and molecular signs of neurodegeneration, including parkinsonism.

Genetic defects in several other autophagy components have already been linked to neurodegenerative disorders, indicating that well‐maintained autophagic flux associated with normal EPG5 activity is crucial for the normal function and maintenance of the central nervous system.^7^, ^36^, ^37^, ^38^, ^39^ A widely acknowledged hypothesis regarding the “atypical form” of parkinsonism, an emerging important presentation within the parkinsonian spectrum characterized by rapid progression and cognitive decline, is that massive protein and organellar accumulation may lead to excessive neuronal waste and degeneration.^40^ Previous reports identified autophagopathies, such as WDR45 defects, as the cause of both childhood NDD^41^ and early adulthood parkinsonism,^42^ suggesting substantial clinico‐pathological overlap reflective of the molecular interactions between EPG5 and WDR45 in the autophagosome–lysosome fusion machinery. We argue that onset of a neurodegenerative phenotype in a patient with previous neurodevelopmental presentation should alert clinicians to a neurometabolic disease that may include EPG5 defects, in particular, if more subtle variations of previously recognized *EPG5*‐related features are present, including callosal dysgenesis/thinning as a milder variant of the callosal agenesis typically seen in *EPG5*‐related Vici syndrome.

Complementary to our novel clinical findings, we also further explored the causative nature of the most common recurrent *EPG5* missense variant, in a novel knock‐in mouse model. Epg5 knockout mice have been shown to display some phenotypic similarities with VS patients, including corpus callosum changes and myopathy. However, many features of VS, including facial dysmorphism, cataracts, and hypopigmentation, are not evident in mice, which instead develop a very aggressive form of selective neuronal degeneration.

Compared with the previously published *Epg5* knockout mouse model, our *Epg5* ^*Q331R*^ mouse showed a milder phenotype with age‐dependent motor features, corresponding to the human phenotypes associated with *EPG5* missense variants.^21^ Interestingly, the phenotype in the *Epg5* ^*Q331R*^ mouse suggested an age‐dependent neurological motor coordination impairment rather than a primary neuromuscular presentation, again corresponding to the novel phenotypes reported here in humans.

We could also demonstrate that the autophagy defect in our knock‐in model affects different central nervous system regions selectively, in a pattern consistent with the selective central nervous system involvement seen on brain imaging in humans with *EPG5*‐RDs. Although reduced stride length and imbalance in *Epg5* ^*Q331R*^ mice resemble phenotypes observed in mouse models of MPTP‐induced parkinsonism,^43^ we were unable to fully investigate any phenotypic worsening because, as according to UK regulations, mice had to be euthanized at ~12 months‐of‐age for their recurrent spontaneous and severe seizures, as previously reported.^15^ However, a putative link between *EPG5* and parkinsonism is also supported by our previous observation of a specific age‐related loss of dopaminergic neurons in an *epg5* knockout *Drosophila melanogaster* model,^15^ and of a hypokinetic locomotion disorder in a *C. elegans epg‐5* knockdown model reported in the present study.

In *C. elegans*, we also observed an increase in spare oxidative capacity with knockdown of *epg‐5* and other genes involved in autophagosome–lysosome fusion (*rab‐7/RAB7*, *ccz‐1/CCZ1*), and of the mitophagy gene *pdr‐1/PRKN* when compared with controls. An increased spare capacity has been previously demonstrated in *C. elegans* models of Parkinson's disease, where it was associated with enhanced fusion of mitochondrial networks at L4 larval worm stage^44^; and in lymphoblasts from sporadic Parkinson's disease patients independent of age.^45^ In vivo flux measurements in *epg‐5i* worms showed increased LGG‐1/LC3:RFP autophagosome punctae^46^ and DCT‐1:GFP mitophagosome punctae indicative of stalled selective mitochondrial clearance. As PDR‐1/PRKN positively regulates mitophagy and interacts with the key mitophagy protein DCT‐1/BNIP3,^18^ not unexpectedly we observed impaired mitophagic clearance in knockdowns of either *pdr‐1/PRKN* or *epg‐5/EPG5*, ultimately resulting in perturbed locomotion. We argue that the adolescent‐onset parkinsonism seen at the milder end of the *EPG5*‐related spectrum may be a result of defective mitochondrial clearance considering that monogenic disorders in mitophagy genes show similar dystonia‐parkinsonism phenotypes.

Considering that motor impairment and mitochondrial dysfunction arose from knockdown of any of the genes involved in autophagosome‐lysosome fusion (*epg‐5/EPG5*, *rab‐7/RAB7*, *ccz‐1/CCZ1*) or mitophagy (*pdr‐1/PRKN*), we recognize that *epg‐5/EPG5* disorders are unlikely to be exclusively driven by defective mitophagy: EPG5 defects lead to defective autophagosome–lysosome fusion, thereby impairing removal of all proteins and organelles with numerous consequences that may exert additional pathogenic effects other than impaired mitochondrial clearance; further studies will be required to identify how EPG5 defects lead to dysfunctional removal of other cellular cargoes, including, but not limited to, lipids, peroxisomes, and/or infectious agents.

In conclusion, our findings identify a lifetime continuum of disease related to EPG5 deficiency in humans, and highlight a significant genetic overlap between (ultra)rare neurodevelopmental diseases and more common age‐related neurodegenerative presentations.

## Author Contributions

Ho.S.D., C.D., K.S., R.M., M.R.D., A.dA., H.H., M.F., and H.J. contributed to the conception and design of the study; Ho.S.D., C.D., K.S., R.M., Z.S., A.L.K., N.J.H., K.P.S., P.S., F.B., L.H., B.G., M.Z., C.A., At.S., Ha.S.D., M.S., A.L., M.H., Al.A., Sa.S., R.A., H.G., G.S., Alir.S., J.Z., D.C., D.M., R.D., A.B., G.C., J.A.R., C.M., D.S., S.C., Su.S., N.K., S.P., Af.S., D.E.F., B.E.C., I.J.C., E.B., R.P., A.P.F., B.L., L.A., F.D., Ma.B., F.E., K.J., S.N., D.Y., H.M., Amy.K., A.D.K., C.M., B.K., V.L., K.O., K.D., F.M.C.V.B., Ar.S., Sas.K., S.V., M.Y.K., Ch.B., M.A., G.E.G., L.M., M.M., F.H.G., S.A., N.N., M.N., Sae.K., Ar.K., S.J., R.S., N.A.H., P.O.K., A.C., M.J., Cl.B., K.E.S., M.W., T.S.B., H.T., An.S., S.V., S.H., Ali.S., R.B., N.H., Me.B., N.S.A., J.L., T.A.B., P.M., D.W., J.I.G., E.S., Amn.K., R.F.A., W.E., M.E., M.L., B.S., Y.L., G.Y., B.W., M.H.G.M., D.K., N.E.M., S.B., M.K., E.A., E.S., Y.J., E.G.K., Y.W.S.C., I.K., G.Z., P.B., W.K.C., J.R.L., M.A.K., J.D., J.C.V.K.R., T.K., M.W., C.Y., A.R., R.C., C.D.V., M.G., M.R.D., A.A., H.H., M.F., and H.J. contributed to the acquisition and analysis of data; Ho.S.D., C.D., K.S., R.M., M.R.D., A.A., H.H., M.F., and H.J. contributed to drafting the text or preparing the figures.

## Potential Conflicts of Interest

Nothing to report.

## Supporting information


**Supplementary File 1‐2.** Supporting Information.

**Supplementary File 3.** Supporting Information.

**Supplementary File 4.** Supporting Information.

**Supplementary File 5.** Supporting Information.

**Supplementary File 6.** Supporting Information.

**Supplementary File 7.** Supporting Information.

## Data Availability

Anonymized data is are upon reasonable request according to local ethics committee guidelines.
