# Supplementary material for: Mutations in the Key Autophagy Tethering Factor EPG5 Link Neurodevelopmental and Neurodegenerative Disorders Including Early‐Onset Parkinsonism
Source: Ann Neurol. 2025 Oct 6;98(5):932–50. doi: 10.1002/ana.78013 (PMC12577676; doi:10.1002/ana.78013)
Supplement: Supplementary file 1 — ‐2. Supporting Information. [file ANA-98-932-s002.docx]

**Mutations in the key autophagy tethering factor EPG5 link neurodevelopmental and neurodegenerative disorders including early-onset parkinsonism**

**Supplementary Material**

**Supplementary File 1: Additional methods and results.** Supplementary methods include statistical analysis, protein modelling, and behavioural analyses of the *Epg5* Q331R knock-in mouse model. Methods for *Caenorhabditis elegans* assays include neuronal morphology analyses, locomotion analyses, and oxygen consumption assays. Methods for cellular assays include immunoflourescence, immunoblotting, generation of a GFP-Parkin and mt-Keima expressing stable cell line in MEFs, transmission electron microscopy (TEM), and mitophagy measurement using FACS. Supplementary results include data on protein modelling of EPG5 variants.

**Supplementary File 2: Proforma used for data capture.** Blank clinical proforma with 207 items regarding phenotypic and genomic features of cohort patients. Y = yes, N = no, ND = not defined by collaborator, NA = information not available.

**Supplementary File 3: Main clinical findings in our cohort of patients with *EPG5*-related disorders.** The data from patients show the general information, family history (FH), molecular genetic variant, initial presentation, development, learning behaviour and psychosocial features, cardiac involvement, epilepsy, CNS involvement, movement disorder, muscle involvement, hearing involvement, and references (if previously published).

**Supplementary File 4: Summary of in silico assessments of the *EPG5* missense variants identified in our cohort.** Ensembl Variant Effector Prediction analyses of 147 variants in *EPG5*: location according to GRCh38; consequence (splice, intron, missense, truncating variant); impact according to VEP (high, moderate, low); gene symbol (EPG5); exon number; intron number; HGVSc annotation; HGVSp annotation; cDNA position; CDS position; protein position; amino acid residue; codon annotation; SIFT rank score; PolyPhen rank score; CADD_PHRED score; BayesDel addAF rankscore; BayesDel noAF rankscore; ClinPred rank score; FATHMM converted rank score; GERP++ RS rank score; LRT converted rank score; M-CAP rank score; MPC rank score; MVP rank score; MetaLR rank score; MetaSVM rank score; MutPred rank score; MutationAssessor rank score; MutationTaster converted rank score; PROVEAN converted rank score; PrimateAI rank score; REVEL rank score; gMVP rank score; SpliceAI delta score acceptor gain; SpliceAI delta score acceptor loss; SpliceAI delta score donor gain; SpliceAI delta score donor loss; ADA score; RF score; gnomAD v3 genomes heterozygous allele count; gnomAD v3 genomes allele frequency; gnomAD v3 genomes allele number; gnomAD v3 genomes homozygous allele count; existing variation in dbSNP; ClinVar clinical significance annotation; ClinVar ID.

**Supplementary File 5:** (A) The murine *Epg5^Q331R^* mutation (corresponding to the human *EPG5* p.Gln336Arg variant) causes splicing defects that result in two incorrectly spliced *Epg5* isoforms. Normally spliced *Epg5* containing the point mutation is also present. (B) Above, chromatogram of an *Epg5* p.Gln331Arg/Q331R KI mouse sample. The dominant sequence is shown by the larger peaks and top sequence, which after the mutation corresponds to the beginning of intron 2. The sequence of the smaller peaks is shown by the bottom line, which after the mutation corresponds to the beginning of exon 3. Below, schematic presentation of the third, canonically spliced isoform of *Epg5*. The red arrows indicate the location of the Q331R mutation. (C) *Epg5* KI mice do not show changes in body weight at early stage (1.5 months) or endstage (~12 months). (D) Muscle strength of mutant mice was not affected at endstage when measured in a grip strength assay, suggesting a neurological rather than a neuromuscular basis for the observed abnormalities. (E) All mice show reaching behaviour in the trunk curl test. (F) No head-bobbing was observed in any of the mice. (G) No difference in DPOAE threshold or (H) ABR threshold was detected between wildtype and *Epg5* KI mice. (I) Only 14.28% of the *Epg5* KI mice show a Preyer reflex in the acoustic startle test. (J) *Epg5* KI mice have a later turning response in the contact righting test. Data are presented as percentage in (D,E,H) and mean ± S.E.M. in (D,E,G). Data were analysed using multiple unpaired Student’s t-test. N=5-10 per group. **** = p<.0001.

**Supplementary File 6**: PET-CT in patient 79,1 showed pronounced hypometabolism in the parietal and right occipital lobes, in the posterior gyrus cingulate, and less pronounced also on the left side which was compatible with a severe synaptic dysfunction in these brain regions, as well as a slightly upregulated striatal metabolism.

**Supplementary File 7:** (A) Representative images of mRFP-GFP-LC3 transfected fibroblasts treated with rapamycin and/or bafilomycin for 24 h. Patient fibroblasts show increased accumulation of mRFP and GFP positive puncta (autophagosomes) in untreated and rapamycin condition. Scale bar: 10 µm. (B) Quantification of both RFP and GFP positive (yellow puncta, autophagososmes) and only RFP positive (red puncta, autolysosomes) LC3 puncta from the autophagy flux assay in A. Each experiment examined 20 transfected cells. (C) Immunostaining of healthy fibroblasts with outer mitochondrial marker Tom20, EPG5 and LAMP1 proteins, showing colocalization of EPG5 to fragmented mitochondria (colocalization mask) in response to oligomycin and antimycin-induced mitochondrial membrane depolarization. (D) To check the phopho-Ubquitylation (Ub^p-S65^) of mitochondria under OA condition, control and Q336R fibroblasts were immunostained for Ub^p-S65^, TOM20 and LAMP1. (E) and (F) Colocalization of TOM20, Ub^p-S65^ and LAMP1 was analysed from n ≥ 20 immunostained cells. A significant decrease in colocalization events of LAMP1 and Ub^p-S65^ (indicated by arrowheads) is seen in Q336R fibroblasts with swollen mitochondria. Scale bars, 20 µm and 10 µm, inset, 5 µm. Data represents the mean ± s.d. of at least three independent experiments and analysed by one-way ANOVA with Tukey’s multiple comparisons test. * = p<.05, ** = p<.005, *** = p<.001, **** = p<.0001. ns: not significant.

**Supplementary File 1**

**Methods**

**Molecular modelling of *EPG5* missense variants**

A composite molecular structural model of human EPG5 was generated by determining a three-dimensional density map by single-particle cryo-EM and fitting predicted structural models of three EPG5 domains (hook, finger, shaft) predicted by AlphaFold2 ^1^ into this map. After manually reconnecting the domains, real space refinement was carried out using PHENIX ^2^ to correct for stereochemical errors of the full EPG5 structural model. To model different EPG5 disease variants, the mutagenesis function in the Pymol package version 2.5.5 (Schrodinger, LLC)  was used to identify a side chain rotamer that minimizes clashes with surrounding residues.

**Behavioural analyses of Epg5 Q331R knock-in mouse model.**

Behavioural analysis of mice was performed as described in detail previously ^3^. Staging of the mice was based on their performance in the rotarod assay, where mice were placed on a rotating rod for 5 minutes which accelerated from 4 to 40 rotations per minute and their latency to fall off the rod was measured. Rearing behaviour was observed and manually scored when the mice were allowed to roam around an open arena for 10 minutes. Virtually dividing the open arena in a centre zone and outer zone allowed us to measure their velocity in a specific zone of the arena. Muscle strength was measured by allowing the mice to grip onto a grip strength meter using front and hind paws together. The time it took to right themselves to an upward position was also measured when the mice were positioned on their back.

Auditory brainstem responses (ABRs) were measured using the method described in detail previously^4^. Briefly, mice were anaesthetised and placed in a sound-attenuating chamber (IAC Acoustics Limited) on a heating blanket. Subcutaneous recording needle electrodes (NeuroDart, Unimed Electrode Supplies Ltd) were inserted on the vertex of the head and behind both ears. Responses were evoked by calibrated broadband click stimuli (10 μs duration) and tone pips (5 ms duration, 1 ms onset and offset ramp) at increasing frequencies, at levels ranging from 0–95 dB SPL (in 5 dB steps) at a rate of 42.6 stimuli per second. Those responses (potentials) were then amplified, digitised, and bandpass-filtered between 300–3000 Hz, using custom software and hardware. Thresholds of ABRs were defined as the lowest stimulus level to evoke a visually identifiable waveform.

Distoriton Product Otoacoustic Emissions (DPOAE) were measured in the ear canal of the mice in response to sounds of two combined frequencies (f1 and f2) as described previously^4^. Measurements were made inside a sound-attenuating chamber (IAC Acoustics Limited) with the mouse positioned on a heating blanket. Mice were anaesthetised and were placed in a position where the head was tilted approximately 45 degrees so that the left ear was uppermost. A DPOAE probe containing a microphone and speaker drivers to generate tones was positioned vertically towards the opening of the ear canal. Continuous stimuli at increasing f1 and f2 frequencies were generated and presented using the probe. The f2 tone was presented at a frequency of 1.2 x f1, and a level 10 dB SPL lower than f1. Sound pressure levels of the f2 stimulus ranged from -10 dB to 65 dB in 5 dB steps. The amplitude of the DPOAE was then recorded during stimulus presentation and was digitised for analysis purposes. Several parameters were calculated such as the DPOAE amplitude, the mean noise-floor amplitude, and the standard deviation (SD) of the noise-floor mean. The threshold of the DPOAE was defined as the lowest stimulus level when the DPOAE amplitude exceeded the SD two times above the recording noise-floor.

To measure the acoustic startle reflex, a sound was presented to the mice and their response was observed which was defined by the presence or absence of a brief ear flick. The response was observed by a blinded observer (NI) and either noted as present or absent.

In the trunk curl test the mice are held up by their tail above a horizontal surface. Whether the mouse reaches out to the surface or whether it curls its trunk towards its tail is observed. The response was observed by a blinded observer (NI) and either noted as reaching or curling.

For the contact righting test single mice are placed in a Perspex tube (3 cm in diameter). This way the animal’s four feet are in contact with the bottom of the tube and its back is in contact with the upper side of the tube. The tube is then inverted so the animal is turned upside down. The time it takes the mouse to re-orientate itself in the tube is measured.

**Locomotion analyses in *C. elegans***

RNAi (*luci* control, *epg-5i*, *rab-7i*, *ccz-1i*, and *pdr-1i*) was carried out from egg-on and worms were collected at day 1 adulthood. Swimming behavior and locomotion were analyzed in n = 10 worms for each of three biological replicates using the *C. elegans* swim tracking software CeleST**.**^5^ Stretch was measured as the maximal differences in curvature that occurred between the two most extreme curvature scores at any animal body part during a swimming stroke.^5^ Curling was measured as the relative percentage of time that an animal spent bent around its own body^5^.

## Oxygen consumption assay in *C. elegans* *epg-5* dysfunction

All experiments were performed with worms grown on RNAi bacteria for *luci* control, *epg-5i*, *rab-7i*, *ccz-1i*, and *pdr-1i* from egg-on to day 1 adulthood. Approximately 15 nematodes were picked by hand into each well of a 96-well Seahorse utility plate filled with M9 buffer. Three replicates from independent plates were used in one assay and the assay was performed twice, each on a different day. At least four wells per assay were used as blanks. For determining the maximum oxygen consumption rate and inhibiting mitochondrial respiration, FCCP and sodium azide were used at final concentrations of 15 µM and 10 mM, respectively. Basal and maximum oxygen consumption rate were measured using the Seahorse Respirometer (XFe96)45 at 25°C.

**Immunofluorescence**

Fibroblasts grown on coverslips, either untransfected or transfected with mCherry-Parkin (Addgene plasmid #59419) were treated with OA and fixed with 4% paraformaldehyde and permeabilised with 50 µg/ml digitonin in PBS for 10 min. Cells were then washed, blocked with 3% BSA and incubated with the following primary antibodies: EPG5 (ab122186), Citrate synthase (ab96600) Tom20 (sc-17764), LAMP1 (sc-20011), Phospho-Ubiquitin (Ser65) (#62802) in 3% BSA for 1h at RT followed by incubation with Alexa Fluor 488/594/633- conjugated secondary antibody for 1h at RT. Coverslips were mounted on glass slides and imaged as described in Materials and Methods. Mitophagy events/cell was quantified in Fiji by counting the mask of the colocalized regions from Parkin-CS mask and LAMP1 mask. 3D reconstruction was carried out using the Surface plugin of Imaris 8.4.2 (Bitplane). The Pearson coefficient index (R value) was quantified using Coloc 2 plugin in Fiji.

**Immunoblotting**

Fibroblasts were treated with DMSO, rapamycin (100 nM), bafilomycin (200 nM), oligomycin (2.0 µM) and antimycin A (2.0 µM) for the indicated time. Following treatment, cells were collected and homogenized in RIPA lysis buffer containing freshly added complete EDTA-Free protease (Roche) and Phosphatase Inhibitor Cocktail 2 (Sigma-Aldrich). All lysates were centrifuged and quantified using the Pierce BCA Assay Kit (Thermo Scientific #23227). Samples were separated on 12% Bis-Tris gels (#NW00122) immersed in MOPS running buffer (Invitrogen #NP0001) and transferred onto PVDF membranes (1620175, Bio-Rad) and subsequently probed with the following antibodies for protein detection: SQSTM1/p62 (Abcam, ab56416), CALCOCO2/NDP52 (Thermo Scientific, P422), LC3B ( Sigma, L7543), PINK1 (Abcam, ab23707), Phospho-Ubiquitin (Ser65) (Sigma-Aldrich, ABS1513-I), pan Ubiquitin (Santa Cruz Biotechnology, sc-166553), β-Actin (Cell Signaling,3700), HRP conjugated anti-rabbit and anti-mouse (Jackson ImmunoResearch, # 111-035-045, # 315-035-045). After incubation with secondary antibody, PVDF membranes were imaged by the BIO-RAD ChemiDoc XRS imaging system. Densitometry analysis was performed using Fiji.

**Generation of GFP-Parkin and mt-Keima expressing stable cell line in MEFs**

MEFs transfected with GFP-Parkin were cultured and selected in DMEM supplemented with G418 (500 μg/ml) until stable clones were visible. The stable clones were transferred to 96 well plate to obtain single clones using serial dilution method. These GFP-parkin single clones were used for TEM studies. To generate mt-Keima expressing MEFs, GFP-Parkin stable cells were transduced with pCHAC-mtmKeima-IRES-MCS2 (Addgene plasmid #72342) retrovirus for 24 h and selected for protein expression using fluorescence sorting.

**Transmission electron microscopy (TEM)**

GFP-Parkin MEFs cultured on coverslips and treated with OA to induce PINK/Parkin-dependent mitophagy. MEFs were co-treated with bafilomycin (Baf A) during OA-induced mitophagy to prevent mitophagosome turnover and thereby enable comparisons of mitophagosome number and size. After treatment, coverslips were fixed in EM fixative (2% glutaraldehyde + 2% paraformaldehyde in 0.1M sodium cacodylate) for 1 hr followed by washings with 0.1M Cacodylate Buffer. The Coverslips were then fixed in 1% osmium tetroxide and 1% potassium ferricyanide in 0.1M sodium cacodylate, followed by sequential dehydration in ethanol. The coverslips were embedded in Epoxy Resin (Araldite CY212) Kit (Agar Scientific Ltd.) according to the standard protocol. Embedded coverslips were sectioned to 50nm using an ultra-microtome fitted with a diamond knife, mounted onto TEM compatible copper grids. The grids were then stained with Lead Citrate for 3 min before proceeding for Imaging. Images were acquired using Jeol 1400 Transmission Electron Microscope. Images were taken at a magnification between 800X and 1200X (digital magnification).

Mitophagososmal structures were segmented using DeepMIB software^6^. To quantify mitophagosome area and number, images were imported in Fiji. Mitophagosome structures were manually traced to determine the area. To quantify mitophagosome numbers per square micron, a 16 × 16 μm grid was overlaid on each image to quantify mitophagosomal structures which included 90% of cytoplasmic area, and the numbers were presented per μm2 of cytoplasm.

**Mitophagy measurement using FACS**

MEFs stably expressing GFP-Parkin and mt-Keima treated as indicated and then resuspended in sorting buffer (145 mM NaCl, 5 mM KCl, 1.8 mM CaCl_2_, 0.8 Mm MgCl_2_, 10 mM HEPES, 10 mM glucose, 0.1% BSA). Measurements of mt-mKeima signal were made using BD LSRFortessa™ X-20 flow cytometer equipped with a 405-nm and 561-nm laser. Cells were excited with a violet laser (405 nm) with emission detected at 610 ± 10 nm with a BV605 detector and with a yellow-green laser (561 nm) with emission detected at 610 ± 10 nm by a PE-CF594 detector simultaneously. For each sample, 20,000 events were collected and gated for mt-mKeima positive cells using FACSDIVA software (v8.0.1). Data were analysed using FlowJo (v10, Tree Star).

**Mitochondrial oxygen consumption rate (OCR)**

Measurements of OCR was performed with the Seahorse Bioscience XFe96 bioanalyzer using the Seahorse XF Cell Mito Stress Test Kit (Agilent #103015-100). Wild-type and Q331 MEFs were seeded on XF96 cell culture microplates (Agilent #102416-100) 2 days before the experiment (WT and Q331R MEFs, 1 × 104 cells/well). On the day of the experiment, the culture medium was replaced with Seahorse XF Base medium (Agilent #103334-100) supplemented with 1mM pyruvate (Gibco 11360070), 2 mM glutamine (Gibco #25030081) and 10mM glucose (Gibco #A2494001) and incubated for 30 min at 37 °C in a CO2-free incubator before loading into the Seahorse Analyser. After measuring basal respiration, the drugs oligomycin (2.5 μM), FCCP (1.5 μM, 2.5 μM), and rotenone/antimycin A (0.5 μM/ 0.5 μM) were added to each well in sequential order. Data were analysed using the XF Cell Mito Stress Test Report Generator. After the assay, cells were stained with Hoechst 33342 (1 μM; Thermo Scientific #62249) for 15 min. ImageXpress was then used to count the numbers of cell nuclei (cell numbers) in each well. The normalisation of the experiments is based on the relative cell numbers obtained.

**RNA isolation and quantitative PCR**

Total RNA was extracted from MEFs using the RNeasy Plus Mini Kit (QIAGEN #74134) according to the manufacturer’s instructions. Quantification was done on the Nanodrop 2000c (Thermo Scientific #ND-2000). cDNA was synthesized using SuperScript III (Invitrogen) and used for quantitative PCRs (qPCRs) using SYBR Green (Invitrogen) on an ABI Prism 7000 sequence detection system (Life Technologies). Data were analyzed using the comparative 2−ΔΔCt method. Ct of the *SNCA* gene was normalized to that of *TUBB*. Primers used are listed as follows: *SNCA*, 5′-ACCAAACAGGGTGTGGCAGAAG-3′ (forward) and 5′-CTTGCTCTTTGGTCTTCTCAGCC-3′ (reverse); *TUBB*, 5′- ATGGACGAGATGGAGTTC -3′ (forward) and 5′- TTGAGTAAGACGGCTAAGG -3′ (reverse).

**Results**

**Protein modelling**

For the p.Arg1293Cys mutation, the protein model shows that the sidechain of Arg1293 is buried within a hydrophobic core. This residue may be involved in hydrogen bond interactions with its neighboring residues. The substitution of Arg with Cys, which has a shorter sidechain, may disrupt these interactions and packing, destabilizing the local structure. For the p.Arg1905Gln mutation, the protein model shows that Arg1905 is located at the tip of a helix adjacent to a flexible loop region. Potential hydrogen bond and hydrophobic interactions may be disrupted upon mutation into a shorter sidechain residue Glu. For the p.Cys2038Arg mutation, the protein model shows that Cys2038 is located in a loop region between two helices, potentially forming a disulfide bond interaction with Cys2084. Substituting Cys with Arg may disrupt the disulfide bond interaction and the positively charged sidechain may destabilize the helical bundle formation. For the p.Trp2097Cys mutation, the protein model shows that Trp2097 is located in a loop region between two helices and is buried within a hydrophobic core. Replacement of Cys may disrupt local interactions and packing in this region. For the p.Met2123Arg mutation, the protein model shows that Met2123 is located in the core of the helical bundle and surrounded by hydrophobic residues. The substitution of Met with Arg, which has a positive charge, may introduce charges that disrupt the local structure.

**References**

1. Jumper J, Evans R, Pritzel A, et al. Highly accurate protein structure prediction with AlphaFold. Nature. 2021 Aug;596(7873):583-9.

2. Afonine PV, Klaholz BP, Moriarty NW, et al. New tools for the analysis and validation of cryo-EM maps and atomic models. Acta Crystallogr D Struct Biol. 2018 Sep 1;74(Pt 9):814-40.

3. Baron O, Boudi A, Dias C, et al. Stall in Canonical Autophagy-Lysosome Pathways Prompts Nucleophagy-Based Nuclear Breakdown in Neurodegeneration. Curr Biol. 2017 Dec 4;27(23):3626-42.e6.

4. Ingham NJ, Banafshe N, Panganiban C, et al. Inner hair cell dysfunction in Klhl18 mutant mice leads to low frequency progressive hearing loss. PLoS One. 2021;16(10):e0258158.

5. Restif C, Ibanez-Ventoso C, Vora MM, Guo S, Metaxas D, Driscoll M. CeleST: computer vision software for quantitative analysis of C. elegans swim behavior reveals novel features of locomotion. PLoS Comput Biol. 2014 Jul;10(7):e1003702.

6. Belevich I, Jokitalo E. DeepMIB: User-friendly and open-source software for training of deep learning network for biological image segmentation. PLoS Comput Biol. 2021 Mar;17(3):e1008374.

**Supplementary File 2**

| **General information** | FamNr |
| --- | --- |
|  | Patient Nr |
|  | Internal ID |
|  | Prev. Pub. Nr |
|  | *Surname* |
|  | *First name* |
|  | Sex |
|  | DoB |
|  | Current age or age at death |
|  | Alive or dead? |
|  | Diagnosis |
|  | Country |
|  | Contributor |
|  | Email |
| **Documentation** | Clinical images (Y/N) |
|  | Previous genetic testing (Y/N) |
|  | DNA available (Y/N) ? |
|  | Where ? (Details/NA/ND) |
|  | MRI available (Y/N) ? |
|  | Where ? (Details/NA/ND) |
|  | Fibroblasts available (Y/N/ND) |
|  | Where ? (Details/NA/ND) |
|  | Muscle biopsy available (Y/N) ? |
|  | Where ? (Details/NA/ND) |
|  | Other biosamples available (specify) ? |
| **References (if previously published) (Patient Nr in publication)** |  |
| **References (if previously published) (Patient Nr in publication)** |  |
| **References (if previously published) (Patient Nr in publication)** |  |
| **Family history (FH)** | Pedigree (Y/N) |
|  | Consanguinity (Y/N) |
|  | Affected relatives (Y/N) (Details) |
|  | FH Vitiligo (Y/N) (Details) |
|  | FH Cancer (Y/N) (Details) |
|  | FH Neurology (Y/N) (Details) |
|  | FH Twin pregnancies (Y/N/ND) (Details) |
|  | FH Other (Y/N) (Details) |
| **Genetics** | Testing (Type/Location) |
|  | Phase (homozygous/heterozygous) |
| ***EPG5* Variant 1** | Exon |
|  | DNA |
|  | Protein |
| ***EPG5* Variant 2** | Exon |
|  | DNA |
|  | Protein |
| ***EPG5* Variant 3** | Exon |
|  | DNA |
|  | Protein |
| **Presentation** | Presentation (Details/ND) |
| **Cause of death** | Cause of death (Details/NA/ND) |
| **Perinatal** | Foetal movements (Reduced/Increased/ND) |
|  | IUGR (Y/N/ND) |
|  | Gestation (Weeks) (ND) |
|  | Birth weight (kg) (Centile) |
|  | Birth length (cm) (ND) |
|  | Birth OFC (cm) (Centile) (ND) |
|  | Neonatal presentation (Detail) (ND) |
| **Development** | Motor development (Normal) (Delayed) (ND) |
|  | Sitting (age) (never) (ND) |
|  | Walking (age) (never) (ND) |
|  | Best motor ability (Detail) (ND) |
|  | Speech development (Normal) (Delayed) (ND) |
|  | Best language Skill (Detail) (ND) |
|  | Hearing (Normal/Abnormal/ND) |
|  | Hearing (Details) (ND) |
|  | Vision (Normal/Abnormal/ND) |
|  | Vision (Details/ND) |
|  | Cognitive Development (Normal) (Delayed) (IQ) (ND) |
| **Somatic development** | Current weight (kg) (ND) |
|  | Current weight (age) (Centile) (ND) |
|  | Failure to thrive (Y/N/ND) |
|  | FTT details (Details/NA/ND) |
|  | Current height (cm) (ND) |
|  | Current height (Age/Centile/ND) |
|  | Current OFC (cm) (ND) |
|  | Current OFC (Age/Centile/ND) |
| **General features** | Dysmorphic features (Y/N/ND) |
|  | Dysmorphic features (Details/ND) |
|  | Hypopigmentation (Y/N/ND) |
| **CNS involvement** | CNS involvement (Y/N/ND) |
|  | Brain MRI (Y/N/ND) |
|  | Brain MRI Age (ND) |
|  | MRI summary (Details/ND) (please attach separate report) |
|  | Callosal agenesis (Y/N/ND) |
|  | Cerebellar atrophy (Y/N/ND) |
|  | Pontocerebellar hypoplasia (Y/N/ND) |
|  | Thalamic involvement (Y/N/ND) |
|  | Heterotopia (Y/N/ND) |
|  | Schizencephaly (Y/N/ND) |
|  | Calcifications(Y/N/ND) |
|  | Iron deposition (Y/N/ND) |
|  | Microcephaly (Y/N/ND) |
|  | Other CNS abnormalities (Y/N/ Details/ND) |
|  | PM Brain (Y/N/ND) |
|  | PM Brain (Details/ND) |
| **Epilepsy** | Seizures (Y/N/ND) |
|  | Seizure onset (Age/ND) |
|  | Seizure Type(s) (Details/ND) |
|  | EEG Findings (Details/ND) (please attach formal report) |
|  | AED (Details/ND) Indicate if effective (E) or ineffective (I) |
|  | Ketogenic diet (Y/N/ND) Indicate if effective (E) or ineffective (I) |
|  | Status epilepticus (Y/N/ND) |
|  | Non-convulsive status (Y/N/ND) |
| **Movement disorder** | Movement disorder (Y/N/ND) |
|  | Movement disorder details (Details/NA/ND) |
|  | Dystonia (Y/N/ND) |
|  | Dystonia Onset (Age/NA/ND) |
|  | Myoclonus (Y/N/ND) |
|  | Myoclonus Onset (Age/NA/ND) |
|  | Spasticity (Y/N/ND) |
|  | Spasticity Onset (Age/NA/ND) |
|  | Parkisonism (Y/N/ND) |
|  | Parkinsonism Onset (Age/NA/ND) |
|  | MD Medication (Details/NA/ND) Indicate if effective (E) or ineffective (I) |
|  | CSF Neurotransmiitter (Normal/Abnormal/ND) |
|  | Other neurological features (Details/N/ND) |
| **Learning, behaviour and psychosocial features** | Cognitive abilities (Details/ND) |
|  | Psychiatric/behavioural abnormality (Details/N/ND) |
| **Muscle involvement** | Muscle weakness (Y/N/ND) |
|  | Muscle weakness details (Facial/Proximal/distal/axial/general/ND) |
|  | CK levels (IU/l/ND) |
|  | Muscle Biopsy (Y/N/ND) |
|  | Muscle biopsy Age (Age/ND) |
|  | Muscle Biopsy Details (Details/ND) Please attach report |
|  | Type 1 predominance (Y/N/ND) |
|  | Fibre Type disproportion (Y/N/ND) |
|  | Vacuoles (Y/N/ND) |
|  | Cores (Y/N/ND) |
|  | Respiratory chain enzyme (RCE) studies (Y/N/ND) |
|  | RCE details (Details/ND) |
|  | Electron microscopy (EM) (Y/N/ND) |
|  | EM Details (Details/ND) |
| **Nerve involvement** | Neuropathy (Y/N/ND) |
|  | DTR (Absent/Reduced/Increased/ND) |
|  | EMG (Normal/Abnormal/ND) |
|  | EMG Details (Details/ND) |
|  | Nerve biopsy (Y/N/ND) |
|  | Nerve biopsy Details (Details/NA/ND) |
| **Ocular involvement** | Ocular involvement (Y/N/ND) |
|  | Cataracts (Y/N/ND) |
|  | Cataracts Onset (Age/ND) |
|  | Optic atrophy (Y/N/ND) |
|  | Other ocular abnormalities (Details/N/ND) |
|  | VER (Y/N/ND) |
|  | VER Detail (Detail/NA/ND) |
| **Hearing involvement** | Hearing involvement (Y/N/ND) |
|  | Sensorineural deafness (Y/N/ND) |
|  | BAER (Y/N/ND) |
|  | BAER Details (Details/NA//ND) |
| **Cardiac involvement** | Cardiac involvement (Y/N/ND) |
|  | Cardiac involvement Detail (Detail/NA/ND) |
|  | Congenital heart defect (Y/N/ND) |
|  | CHD Type (Type/NA/ND) |
|  | Cardiomyopathy (CM) (Y/N/ND) |
|  | Onset CM (Age/NA/ND) |
|  | Type CM (Type/NA/ND) |
|  | Cardiac US (Y/N/ND) |
|  | Cardiac US Details (Details/ND) |
|  | Cardiac MRI (Y/N/ND) |
|  | ECG (Y/N/ND) |
|  | ECG Details (Details/ND) |
| **Pulmonary Involvement** | Pulmonary involvement (Y/N/ND) |
|  | Pulmonary involvement details (Details/NA/ND) |
|  | Interstitial lung disease (Y/N/ND) |
| **Thyroid involvement (Y/N/ND)** | Thyroid involvement (Y/N/ND) |
|  | Thyroid involvement details (Details/NA/ND) |
| **Thymus involvement (Y/N/ND)** | Thymus involvement (Y/N/ND) |
|  | Thymus involvement details (Details/NA/ND) |
| **Hepatic involvement** | Hepatic involvement (Y/N/ND) |
|  | Hepatic involvement details (Details/NA/ND) |
|  | Hepatomegaly (Y/N/ND) |
| **Renal involvement** | Renal involvement (Y/N/ND) |
|  | Renal involvement details (Details/NA/ND) |
|  | Renal tubular acidosis (Y/N/ND) |
|  | Electrolyte disturbance (Y/N/ND) |
|  | Electrolyte disturbance details (Details/NA/ND) |
| **Gastric involvement** | Gastric involvement (Y/N/ND) |
|  | Feeding difficulties (Y/N/ND) |
|  | Gastrostomy (Y/N/ND) |
|  | Enterocolitis (Y/N/ND) |
| **Immune system** | Immune system involvement (Y/N/ND) |
|  | Immune system involvement details (Details/NA/ND) |
|  | Immunodeficiency (Y/N/ND) |
|  | Immunodeficiency Type (B-cell/T-cell/Combined/ND) |
|  | Age at diagnosis (Age/NA/ND) |
|  | Number of febrile infections within 1st year of life (n/ND) |
|  | Serositits (pleuritis / peritonitis) (Y/N/ND) |
|  | Arthritis/uveitis (Y/N/ND) |
|  | Meningitis/ encephalitis (Y/N/ND) |
|  | Lymphadenopathy (Y/N/ND) |
|  | Allergies/eczema (Y/N/ND) |
|  | Measles-like rash (Y/N/ND) |
|  | Ectodermal dysplasia (Y/N/ND) |
|  | Ichthyosis (Y/N/ND) |
|  | Ear infections (Y/N/ND) |
|  | Skin infections (Y/N/ND) |
|  | Other infections (Details/NA/ND) |
|  | Amyloidosis, hyper-IgE (Y/N/ND) |
|  | Immunoglobulin results (Details/ND) |
|  | Inflammatory markers (Details/ND) |
| **Haemotology** | Haematological abnormalities (Y/N/ND) |
|  | Haematological abnormalities details (Details/NA/ND) |
|  | Anaemia (Y/N/ND) |
|  | Neutropenia (Y/N/ND) |
|  | Lymphopenia (Y/N/ND) |
|  | Thrombocytopenia (Y/N/ND) |
|  | Other haematological abnormalities (Details/NA/ND) |
| **Therapies trialled** | Therapies trialled (Details/NA/ND) |
| **Additional information** | Additional information 1 |
|  | Additional information 2 |
| **Date entry** | Date entry (Date/Initial) |
