# Supplementary figures and images for: Mutations in the Key Autophagy Tethering Factor EPG5 Link Neurodevelopmental and Neurodegenerative Disorders Including Early‐Onset Parkinsonism
Source: Ann Neurol. 2025 Oct 6;98(5):932–50. doi: 10.1002/ana.78013 (PMC12577676; doi:10.1002/ana.78013)

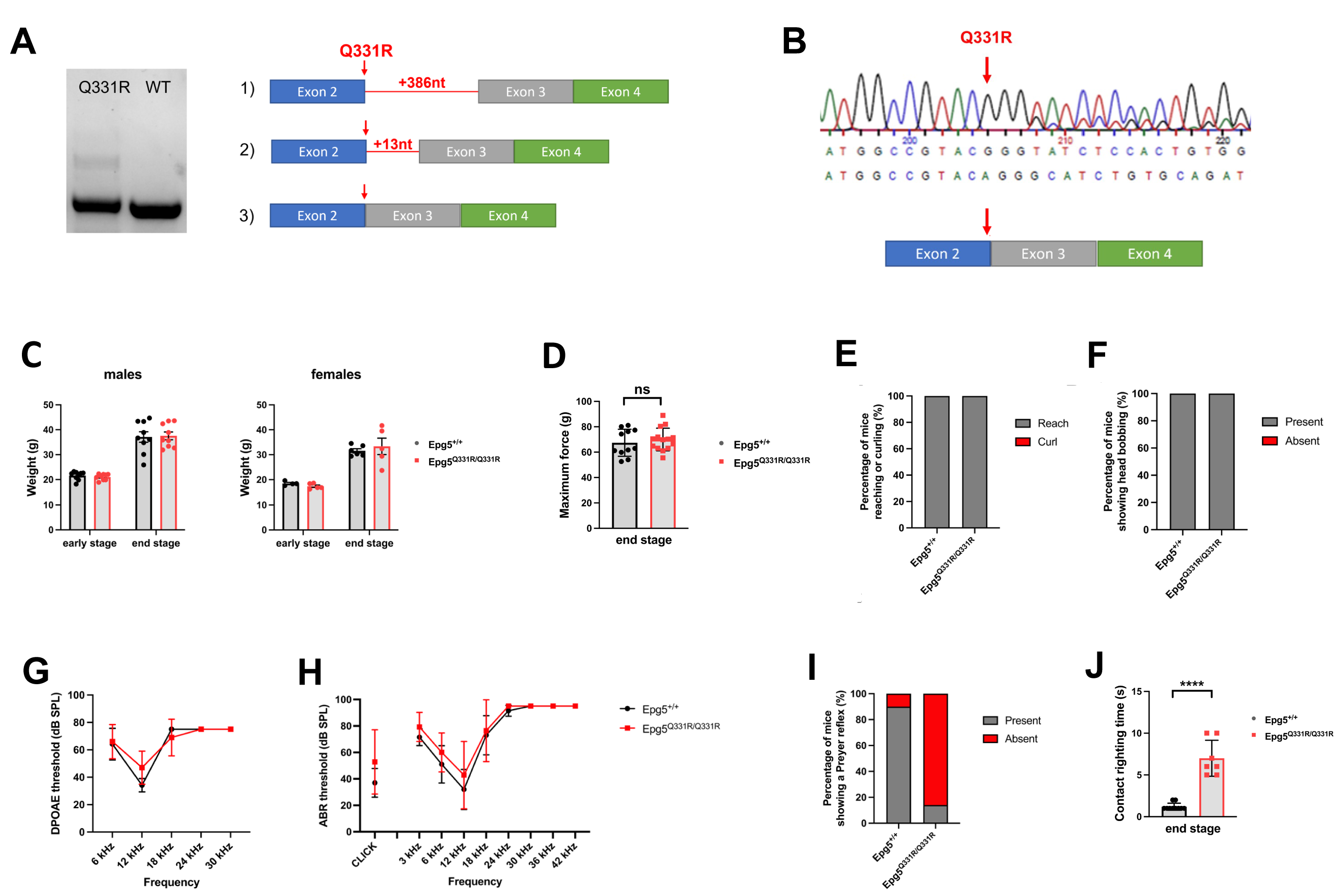

Supplement: Supplementary file 4 — Supplementary File 5. Supporting Information. [file ANA-98-932-s005.tif]

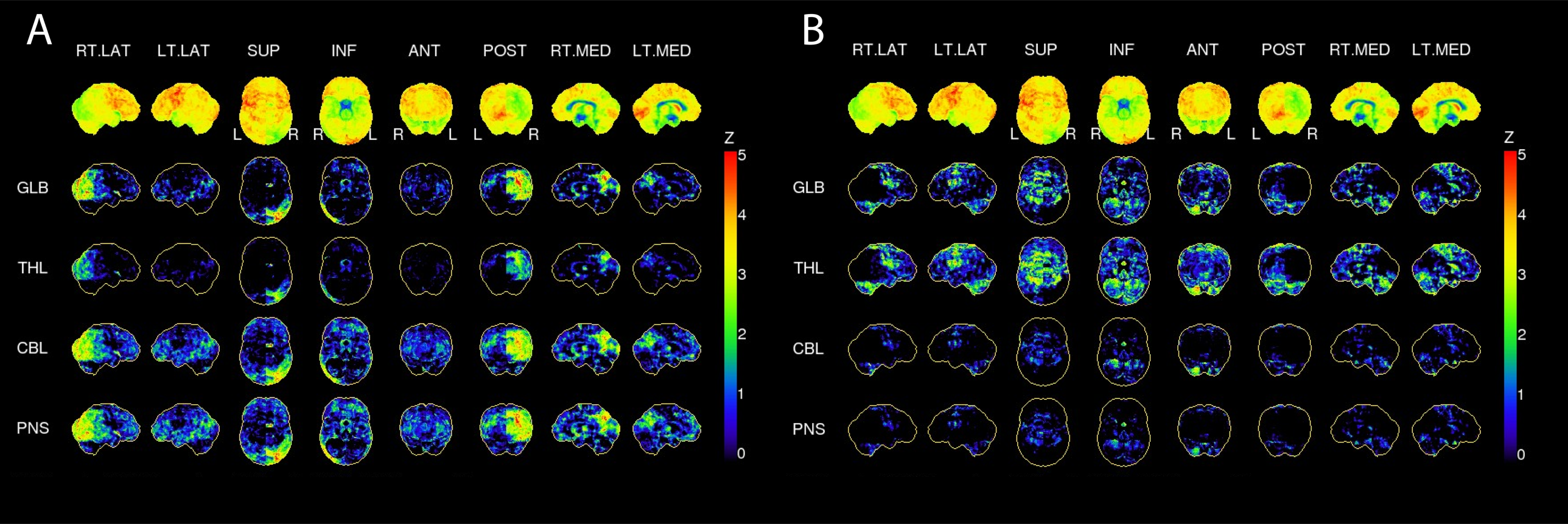

Supplement: Supplementary file 5 — Supplementary File 6. Supporting Information. [file ANA-98-932-s001.tif]

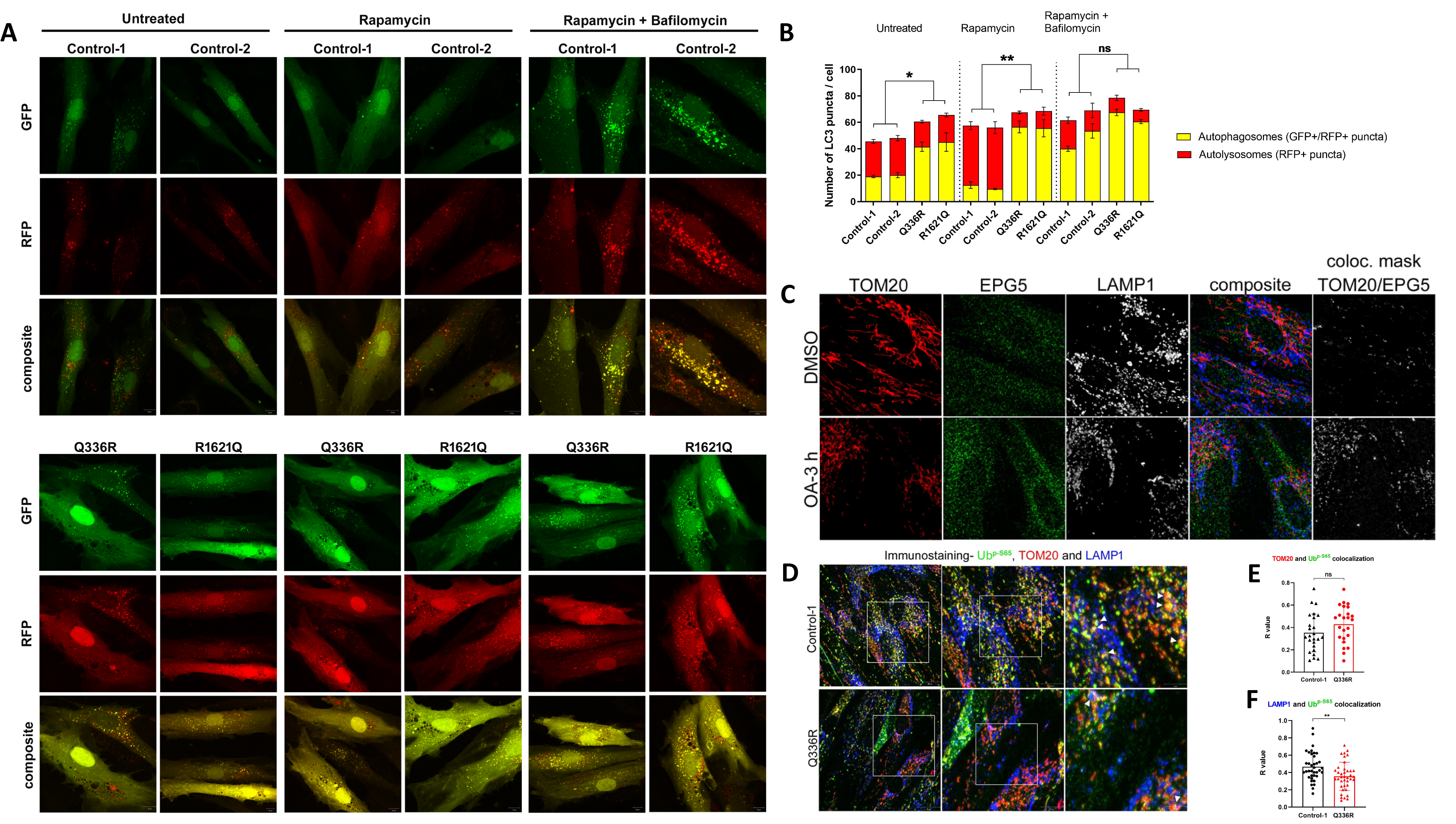

Supplement: Supplementary file 6 — Supplementary File 7. Supporting Information. [file ANA-98-932-s003.tif]
